# A Tailored Phospho‐p53 Library Probes Antibody Specificity and Recognition Limitations

**DOI:** 10.1002/cbic.202500256

**Published:** 2025-06-23

**Authors:** Mateusz Hess, Jonathan H. Davies, Sofia Margiola, Sonja Schneider, Thomas Hicks, Nishant Rai, Manuel M. Müller

**Affiliations:** ^1^ Department of Chemistry King's College London Britannia House, 7 Trinity Street London SE1 1DB UK

**Keywords:** antibodies, p53, peptide synthesis, phosphorylation, post‐translational modifications, protein modifications

## Abstract

The tumor suppressor protein p53, known as the “guardian of the genome,” is regulated by a complex network of post‐translational modifications. Phosphorylations at 7 Ser/Thr residues within the N‐terminal transactivation domain 1 (TAD1) play a role in p53 activation, yet their precise mechanisms of action remain elusive due to challenges in accessing well‐defined phosphorylated isoforms. To address this limitation, this study harnesses a recently developed approach for the semisynthesis of site‐specifically phosphorylated p53 to generate a comprehensive library of singly phosphorylated p53 including all TAD1 sites: Ser6, Ser9, Ser15, Thr18, Ser20, Ser33, and Ser37. The library was then used to probe the specificity of common p53 antibodies in western blot analysis. This study's results confirm the specificity of the target site of most phosphorylation‐specific anti‐p53 antibodies, but also reveal wide‐spread epitope masking by phosphorylation, which has implications for p53 research and diagnostics. This "designer" p53 library thus provides a toolkit to study the function of p53 phosphorylation directly and indirectly as a quality control agent for some of the most widely used reagents in the field.

## Graphical abstract

Site‐specifically phosphorylated isoforms of the tumor suppressor p53 – encompassing all seven known phosphorylation sites within the TAD1 domain –are created upon optimization of semisynthesis protocols. This “designer" p53 library enabled validation of phosphorylation‐specific p53 antibodies and revealed epitope masking by phosphorylation in commonly used antibodies.

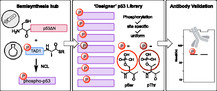

## 1 Introduction

The tumor suppressor protein p53, a critical regulator of cell cycle arrest and apoptosis, is tightly controlled by a complex network of post‐translational modifications (PTMs).^[^ ^1^ ^]^ Among these, phosphorylation plays a crucial role in modulating p53 activity, stability, and interactions with key regulatory partners.^[^ ^2^ ^]^ The N‐terminal transactivation domain 1 (TAD1) of p53 is a hotspot for phosphorylation, harboring seven serine/threonine residues that are dynamically modified in response to cellular stress.

These phosphorylation events have been implicated in various aspects of p53 regulation. Phosphorylation of Ser15 by ataxia‐telangiectasia mutated kinase is the most commonly described modification and is believed to initiate the p53 response to DNA damage.^[^ ^3^, ^4^, ^5^, ^6^ ^]^ Subsequently, p53 is phosphorylated by CK1 on Thr18 which stabilizes p53 by reducing its interaction with the negative regulator MDM2.^[^ ^7^, ^8^, ^9^ ^]^ Phosphorylations at Ser20 and Ser37 have also been shown to enhance p53 activity, in part by enhancing p53's interaction with the acetyltransferases p300 and CREB binding protein.^[^ ^10^, ^11^ ^]^ Closer to the very N‐terminus, phosphorylation of Ser6 and Ser9 has been implicated in transforming growth factor beta signaling and may play a role in stabilization of p53 mediated by the chaperonin system.^[^ ^12^, ^13^, ^14^ ^]^ A more complex picture is presented by phosphorylation of Ser33 which creates a binding site for the phosphorylation‐dependent prolyl isomerase Pin1, in turn enhancing p53 activity.^[^ ^15^, ^16^ ^]^ Despite this knowledge, a complete understanding of how these individual phosphorylation events contribute to p53 regulation remains elusive due to the multitude of p53‐interacting proteins, the interplay between co‐occurring phosphorylation marks, and their potential to trigger accumulation of further PTM signatures throughout the protein.

**Scheme 1:**
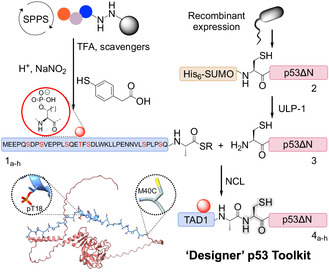
Protein semisynthesis strategy to generate p53 isoforms carrying single‐site phosphorylation marks within TAD1.

Given the complexity of p53 signaling in vivo, in vitro methods are critical to fill in mechanistic details of how PTMs control p53 function. Traditional approaches for generating phospho‐proteins, such as point mutations to Asp or Glu which mimic the negative charge of phosphates or co‐expression with cognate kinases, have often been used in p53 research, providing valuable information on its signaling pathways.^[^ ^17^, ^18^, ^19^, ^20^ ^]^ However, these approaches may fail to accurately recapitulate the effects of single‐site phosphorylation^[^ ^21^, ^22^, ^23^ ^]^ due to imperfect mimicry and lack of kinase specificity. The latter can contribute to heterogeneity of isolated phosphorylated species, although this issue can be rectified with compensatory mutations in competing sites and has been successfully used to investigate phosphorylation‐dependent conformational changes in p53.^[^ ^18^ ^]^ Synthetic chemistry^[^ ^24^, ^25^ ^]^ and biology approaches^[^ ^26^, ^27^ ^]^ which can generate authentic phospho‐proteins are therefore necessary for quantitative structure function studies, and have been adapted to research on p53 phosphorylation^[^ ^28^ ^]^ and other p53 PTMs.^[^ ^29^, ^30^, ^31^ ^]^ Protein semisynthesis via native chemical ligation is particularly powerful because it allows installation of diverse PTMs into relatively large proteins with otherwise minimal perturbations to the target protein.^[^ ^32^, ^33^ ^]^ We have previously developed such a strategy to install site‐specific phosphorylation (at Ser15 and Ser20) into p53 via semisynthesis.^[^ ^28^ ^]^ Here, we extend this method to access a complete library of full‐length p53 variants, each bearing a single phosphate group on a specific serine or threonine residue within TAD1 (see **Scheme** **1**). This approach enables precise control over the site and stoichiometry of phosphorylation, providing a powerful tool to dissect the individual contributions of each phosphosite to the regulation of p53. Additionally, we deploy this unique library to benchmark the specificity and potential shortcomings of commercially available antibodies for detection of p53 and its isoforms.

## 2 Results and Discussion

To generate a comprehensive library of site‐specifically phosphorylated p53 proteins, we first synthesized a series of TAD1 peptides bearing single phosphorylation modifications. Building upon our previous work, where we successfully synthesized TAD1 phosphorylated at Ser15 and Ser20,^[^ ^28^ ^]^ we expanded and improved on our methodology to encompass all seven possible phosphorylation sites within the TAD1 region (residues 1–39). Each singly phosphorylated TAD1 peptide (**1a‐g**) was prepared by Fmoc‐SPPS as a C‐terminal acyl hydrazide (see Scheme 1 for sequence) to facilitate subsequent coupling with the recombinant fragment of p53 via Native Chemical Ligation (NCL).^[^ ^28^, ^32^, ^33^ ^]^ Phospho‐amino acids (pSer/pThr) were incorporated using commercially available monobenzyl‐protected building blocks. Similarly, we prepared a tri‐phosphorylated version (TAD1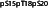, **1 h**) using the same protocol, with higher equivalents of the activated amino acid ester employed during solid‐phase peptide synthesis after the incorporation of each phosphoamino acid.

During the synthesis of the TAD1 phosphopeptide hydrazides, we encountered several challenges that impacted the overall yield. While we previously achieved satisfactory yields for TAD1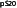,^[^ ^28^ ^]^ the synthesis of TAD1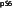, TAD1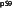, and TAD1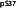 resulted in lower yields of 1%‐2%. We identified the primary issue as a single proline truncation occurring during peptide chain assembly, specifically at the P12‐P13 junction (see Figure S1, Supporting Information). This truncation, arising from inefficient coupling between consecutive proline residues, necessitated multiple rounds of purification and reduced the overall yield. To overcome this hurdle, we employed a pre‐synthesized proline dipeptide (Figure S2–S7, Supporting Information) in the synthesis of TAD1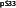 and TAD1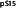. This strategy significantly improved the purity of the crude peptide and increased the final isolated yield to 5% (Table S1 and Figure S8–S15, Supporting Information).

In parallel, we produced p53 lacking TAD1 (p53ΔN) by recombinant protein expression in an *E. coli* host. Conceptually, we followed our previously published procedure.^[^ ^28^ ^]^ p53ΔN was expressed as an N‐terminal His
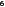
‐SUMO (small ubiquitin‐like modifier) fusion (**2**) using autoinduction media under the lac operator (yielding 60–80 mg of **2** per L of culture). A Cys residue, required for NCL, was introduced by mutating Met40 to Cys. We have previously shown that this ligation site is suitable both from a synthetic perspective and because the M40C mutation is well tolerated in DNA binding and p300 assays.^[^ ^28^ ^]^ The His
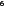
‐SUMO‐p53 fusion protein was isolated and liberated from the tag by proteolytic cleavage with Ubl‐specific protease 1 (ULP‐1), followed by reverse nickel‐nitrilotriacetic acid chromatography. The resulting p53ΔN (**3**) was purified by reverse‐phase high‐performance liquid chromatography (RP‐HPLC) and lyophilized, yielding 10–20 mg of ligation‐ready protein per L of culture. In initial results, we found that the p53 protein is particularly sensitive to oxidation (+16 Da peak in mass spectrum) under denaturing conditions and upon thawing in denaturant. This issue necessitates the use of thoroughly degassed buffers and careful handling to achieve optimal yields, with oxidation below 20%, judged by peak intensity in the mass spectrum (see Figure S16, Supporting Information).

**Figure 1:**
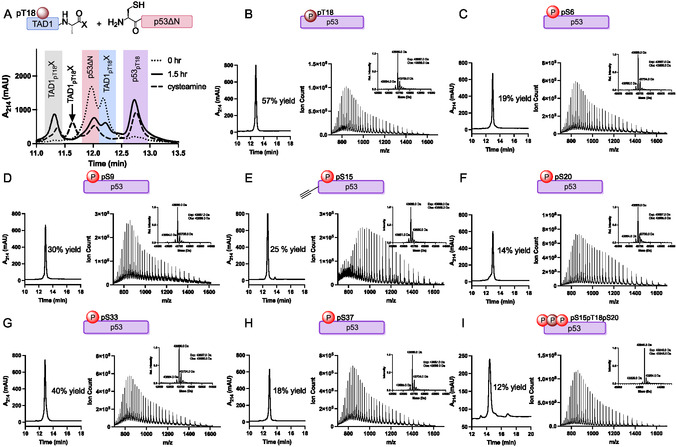
Synthesis of a comprehensive library of TAD1‐mono‐phosphorylated p53 via Native Chemical Ligation. A) RP‐HPLC analysis of the ligation reaction for p53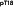 (**4d**), where p53ΔN (**3**) is shaded pink, TAD1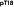 MPAA thioester is shaded blue (*X* = MPAA, **1d**), the ligated product is shaded in violet, hydrolyzed TAD1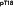 is shaded in gray (*X* = OH), and the quenched TAD1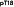 (*X* = cysteamine) is indicated with an arrow. B) RP‐HPLC analysis of purified p53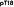 (**4d**) and ESI‐MS analysis of purified p53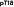 (**4d**). The inset shows the deconvoluted spectrum of the protein product, with minor contributions of −17 Da (deamidation) and +16 Da (oxidation). C–I) RP‐HPLC and ESI‐MS analysis of C) p53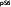 (**4a**), D) p53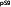 (**4b**), E) p53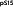 (**4c**), F) p53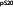 (**4e**), G) p53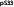 (**4f**), H) p53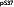 (**4g**), and I) p53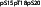 (**4h**).

Next, we optimized ligation conditions. For the NCL reactions, 0.5–1 μmol of TAD1 acyl hydrazides were converted to 4‐mercaptophenylacetic acid (MPAA) α‐thioesters (**1** 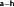) through oxidation with NaNO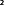 and thiolysis in situ.^[^ ^34^ ^]^ The resulting peptide α‐thioesters were then incubated with 0.25–0.5 μmol of p53ΔN (**3**) at room temperature, observing formation of the full‐length p53 product (**4** 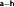) within an hour (**Figure** **1**A and S17–S24, Supporting Information). Using our previously published protocol,^[^ ^28^ ^]^ we achieved 14%, 19% and 12% isolated yield for p53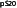, p53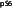 and p53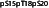, respectively (Figure 1F,C,I, S17, S21, and S24, Supporting Information). Conducting the reaction under argon atmosphere and post‐reaction reduction with 200 mM DTT has increased the yield to 30% for p53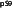 (Figure 1D and S17, Supporting Information). Next, we included 50 mM tris(2‐carboxyethyl)phosphine (TCEP) in the reaction as a reducing agent, which aided reaction analysis via RP‐HPLC by reducing the protein and the MPAA dimer. Furthermore, a post‐reaction treatment with 200 mM cysteamine eliminated secondary peptide‐protein transthioesterification products, as previously observed.^[^ ^28^ ^]^ These interventions (summarized in Figure S25, Supporting Information), together with adjusting equivalents of p53ΔN and TAD1 thioester, have improved our yield to 40% and 57% for p53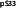 and p53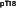, respectively (Figure 1B,G and S22 and S20, Supporting Information). Inclusion of TCEP, although beneficial for reaction monitoring, also led to observable thioester hydrolysis, an observation that was reported for other peptide α‐thioesters.^[^ ^35^ ^]^ We initially hypothesized that increasing the buffer capacity from 50 mM to 200 mM Na
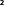
HPO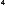 might alleviate the issue, by reducing pH fluctuations in the reaction. However, our results for p53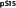 and p53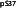 indicate that the use of a high concentration of the buffering agent does not increase the yield of NCL. In our hands, ensuring complete reduction of the N‐terminal cysteine of p53ΔN and a sufficient excess TAD1 thioester led to the best yields.

Antibodies raised against p53 and its PTM isoforms have provided a wealth of information on p53 regulation.^[^ ^36^ ^]^ We therefore performed western blot analysis using commercially available and frequently deployed anti‐p53 and phospho‐specific primary antibodies as a way to cross‐validate our semisynthetic species and the corresponding antibodies.

**Figure 2:**
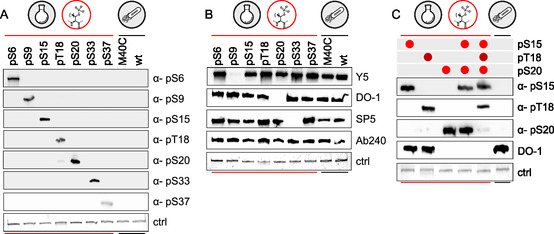
A) Western blot analysis of selectivity of primary antibodies against their phosphorylated p53 target, with 100 ng loading of semisynthetic and unmodified recombinant equivalents; α‐p53(pSer6), abx000209; α‐p53(pSer9), CST #9288; α‐p53(pSer15), Ab223868; α‐p53(pThr18), PA5‐12 660; α‐p53(pSer20), Ab157454; α‐p53(pSer33), Ab75867; α‐and p53(pSer37), Ab182164. B) Impact of p53 phosphorylation on recognition by commonly used α‐p53 antibodies. 50 ng p53 were loaded per lane and blots were probed with DO‐1 (SC‐126), Y5 (Ab32049), SP5 (Ab16665), or Ab240 (ab26). Blocking and incubation with primary antibodies were performed with 3% BSA. C) Epitope masking by proximal phosphorylation marks. 50 ng of p53 variants (with indicated phosphorylation status at Ser15, Thr18 and/or Ser20) were loaded per lane. Blocking and incubation with primary antibodies were performed with 3% BSA (α‐p53(pSer15), Ab223868; α‐p53(pThr18), PA5‐12 660; α‐p53(pSer20), Ab157454; and DO‐1, Ab1101).

Under unoptimized conditions, most antibodies exhibit the expected specificity. Antibodies raised against phosphorylations at Ser6, Ser9, Ser15, Thr18, Ser20, Ser33, and Ser37 displayed selectivity against target p53 isoforms (**Figure** **2**A). Slight cross‐reactivity was observed between the α‐phospho‐Ser20 antibody and p53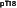. To our surprise, a monoclonal antibody against phospho‐Ser6 (Y179) lacked selectivity toward its target, recognizing all p53 loaded on the blot, compared to its polyclonal equivalent (Figure S26 and S27, Supporting Information). With the notable exception of the monoclonal α‐phospho‐Ser6 antibody, titration of semi‐synthetic p53 variants confirmed the initial determination of specificity (Figure S26, Supporting Information) and a dose‐dependent signal with a linear range up to 100 ng.

We next tested commercial antibodies raised against nonphosphorylated epitopes within the N‐terminal domain of p53 and frequently used to detect total p53 by western blot. We observed that the commonly used p53 antibody DO‐1 suffers from epitope occlusion by Ser20 phosphorylation, which has previously been noted by others in the field.^[^ ^10^, ^36^ ^]^ We report that other anti‐p53 antibodies suffer from epitope occlusion by phosphorylation as well; notably, Y5 was unable to detect p53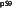, and SP5 failed to detect p53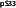 (Figure 2B). As expected, recognition of p53 by Ab240, an antibody raised against the DNA‐binding domain (DBD) of p53, is not disrupted by phosphorylation of the N‐terminal region of p53 (Figure 2B).

We further investigated whether multiple PTMs influence p53 recognition by phospho‐specific and general α‐p53 antibodies. Using a previously characterized p53 isoform phosphorylated at both Ser15 and Ser20,^[^ ^28^ ^]^ the new tri‐phosphorylated variant p53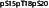 (**4 h**), and various mono‐phosphorylated controls, we observed that additional modifications can perturb antibody recognition (Figure 2C and S29, Supporting Information). For example, detection by the α‐phospho‐Ser15 antibody was negatively affected by the presence of pS20, although, surprisingly, this effect was mitigated by concurrent presence of pT18. The α‐phospho‐Ser20 antibody failed to recognize its target site in p53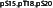, most likely because of the adjacent Thr18 phosphorylation (Figure 2C and S29, Supporting Information). Recognition of both p53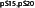 and p53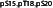 by the DO‐1 antibody was completely lost, as expected based on the observation for the monophosphorylated pS20 isoform (Figure 2C). This result provides further insights into how antibody‐based detection is affected by the PTM state of p53, mirroring similar observations in the context of histones.^[^ ^37^, ^38^ ^]^

## 3 Conclusion

We have generated a library of “designer” p53 that encompasses all known isoforms within TAD1 with single phosphorylation. By incorporating Fmoc‐Pro‐Pro‐OH into our synthetic pipeline, we doubled the yield of synthetic TAD1 peptides and eliminated a challenging side product, thus facilitating purification. Furthermore, we optimized the native chemical ligation protocol, boosting our isolated yield from 14% to up to 57%. This improvement resulted from a combination of factors, including increased peptide thioester equivalents, smaller reaction scales, and the use of TCEP as the reducing agent. Finally, we demonstrated the utility of our semisynthetic phospho‐p53 library by benchmarking the specificity of commonly used antibodies for p53 and its phosphorylated isoforms. Our results reveal that epitope masking by phosphorylation is wide‐spread. Careful selection of antibodies – aided by “designer” p53 isoforms – is therefore critical for obtaining robust results in basic research on p53 and potential diagnostic applications.

## 4 Experimental Section

Detailed descriptions of methods and analytical data are provided in the Supporting Information.

4.1

4.1.1

##### Peptide Synthesis

TAD1 p53 peptide (residues 1–39) were synthesized as C‐terminal acyl hydrazides using established Fmoc solid‐phase methods.^[^ ^28^ ^]^ Peptides were assembled on a hydrazine functionalized 2‐chlorotrityl chloride resin using a combination of automated and manual DIC/Oxyma amino acid couplings (4 eq.). Fmoc deprotection was conducted with 20% piperidine or 5% piperazine in DMF. To prevent oxidation, the N‐terminal methionine was replaced with norleucine or a clickable propargylglycine residue. Phosphoserine and phosphothreonine were incorporated as a mono‐benzyl‐protected building blocks using adjusted coupling conditions detailed in the Supplementary Information. Fmoc‐Pro‐Pro‐OH was included to prevent a ‐Pro truncation. The peptides were cleaved from the resin with cleavage cocktail K, precipitated with cold diethyl ether, and purified by RP‐HPLC. RP‐HPLC and ESI‐HRMS analysis confirmed final peptide purity. Detailed protocols for cleavage, purification, and characterization as well as analytical data are provided in the supporting information.

##### Protein Expression

p53ΔN was expressed in *E. coli* as a His
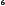
‐SUMO fusion and carried an M4 °C mutation for NCL. The fusion protein was produced in bacterial host grown in an auto‐induction medium (1% tryptone, 0.5% yeast extract, 50 mM NH
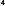
Cl, 25 mM Na
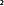
HPO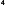, 25 mM KH
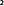
PO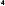, 5 mM Na
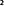
SO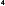, 2 mM MgSO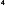, 0.5% glycerol, 0.8% glucose, 0.2% lactose monohydrate, and 100 μg ml^−1^ ampicillin) and isolated as inclusion bodies. The inclusion bodies were solubilized in argon degassed 6 M GdmCl, and the His
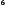
‐SUMO tag was cleaved with ULP‐1 at RT for 1 h in cleavage buffer (150 mM NaCl, 50 mM Tris‐HCl, 1.4 M urea, 100 mM L‐arginine, 0.2 M GdmCl, and 0.75 mM DTT). The resulting p53ΔN protein was then purified using reverse nickel column chromatography. The eluent was treated with 50 mM DTT for 30 min at RT and then precipitated with TCA (100% w v^−1^, 1:4 volume ration of TCA to protein) and centrifuged for 10 min at 7,000 × g at 4 °C. Pellet was redissolved in 6 M GdmCl, desalted, and lyophilized. Lyophilized protein powder was then redissolved in 25% MeCN and injected on preparative RP‐HPLC. RP‐HPLC and ESI‐HRMS were used to verify the purity and identity of p53ΔN intermediate. Detailed protocols for recombinant production, inclusion body preparation, protein cleavage, purification, and characterization as well as analytical data are provided in the Supporting Information.

##### Native Chemical Ligation

TAD1 peptide (≈1 μmol) was dissolved in degassed ligation buffer (6 M GdmCl, 50 mM Na
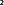
HPO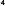, 1.5 mM EDTA, and pH 3). The peptides were then activated by converting them to acyl azides using NaNO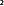 at −15 °C for 15 min. This was followed by adjusting the pH to 7 and reacting with 200 mM MPAA to form the MPAA‐thioester. p53ΔN (0.25–0.5 μmol) was added to the reaction mixture, and the ligation proceeded at room temperature for at least 1 h. In some instances, 50 mM TCEP was added, as described in detail in the supporting information. RP‐HPLC and ESI‐HRMS were used to monitor the progress of the reaction. After 1–1.5 h, the reaction was quenched with 200 mM cysteamine hydrochloride to prevent further reactions. The crude ligation product was then purified using semipreparative RP‐HPLC and analyzed by RP‐HPLC and electrospray ionisation mass spectrometry (ESI‐MS). Specific adjustments to the protocol as well as analytical data for each variant are provided in the supporting information.

##### Western Blot Analysis

Between 50 and 100 ng of semisynthetic or recombinant p53 were resolved by sodium dodecyl sulfate polyacrylamide gel electrophoresis on 10% polyacrylamide gels (containing 0.5% tri‐chloroethanol for stain‐free imaging, unless otherwise noted) and transferred onto polyvinylidene difluoride membranes. Membranes were blocked with 3% bovine serum albumin (BSA), unless otherwise noted, in tris‐buffered saline containing 0.1% Tween 20 (TBS‐T) and subsequently incubated with the appropriate primary antibody in 3% BSA in TBS‐T overnight at 4 °C. Membranes were washed three times with TBS‐T for 5 min and incubated with 1:10 000 HRP‐conjugated secondary antibody (goat anti‐mouse for DO‐1 and Ab240, goat anti‐rabbit for all other antibodies) for 1 h at RT. The secondary antibody was washed away and membranes were imaged on a ChemDoc gel imager (Biorad) following incubation with fresh ECL Clarity Substrate (Biorad).

##### Antibodies

General p53 primary antibodies used: DO‐1, Abcam Ab1101 or SantaCruz SC‐126; Y5, Abcam Ab32049; SP5, Abcam Ab16665; and Ab240, Abcam Ab26. Phospho‐p53‐specific primary antibodies used: α‐p53(pSer6), Abbexa abx000209 or Abcam Ab32132; α‐p53(pSer9), Cell Signaling Technologies #9288; α‐p53(pSer15), Abcam Ab223868; α‐p53(pThr18), ThermoFisher PA5‐12 660; α‐p53(pSer20), Abcam Ab157454; α‐p53(pSer33), Abcam Ab75867; and α‐p53(pSer37), Abcam Ab182164. Antibody dilutions and secondary antibodies are specified in the Supporting Information (Table S2, Supporting Information).

## Conflict of Interest

M.H. and M.M.M. are exploring potential commercial applications of semisynthetic p53.

## Supporting information

Supplementary Material

## Data Availability

The data that support the findings of this study are available in the supplementary material of this article.
