# Supplementary material for: A Tailored Phospho‐p53 Library Probes Antibody Specificity and Recognition Limitations
Source: Chembiochem. 2025 Jun 23;26(14):e202500256. doi: 10.1002/cbic.202500256 (PMC12278346; doi:10.1002/cbic.202500256)
Supplement: Supplementary file 1 — Supplementary Material [file CBIC-26-e202500256-s001.pdf]

Supplementary Information for:

“A tailored phospho-p53 library probes antibody specificity and recognition limitations”

Mateusz Hess, Dr. Jonathan H. Davies, Dr. Sofia Margiola, Sonja Schneider, Dr. Thomas Hicks, Nishant Rai, Dr. Manuel M. Müller*

Department of Chemistry, King’s College London,

Britannia House, 7 Trinity Street, SE1 1DB, London, UK

*To whom correspondence should be addressed; email: manuel.muller@kcl.ac.uk

Index

TAD1 Peptide Synthesis & Characterization ... 3

Materials & Methods ... 3

Chlorotrityl resin functionalization ... 3
General Procedure for SPPS ... 4
Peptide Cleavage and Purification ... 5
Analytical HPLC ... 6
ESI-HRMS of peptides and proteins ... 6
Reverse Phase purification of peptides and proteins ... 6

Truncation -Pro as a challenge in purifications ... 7

Synthesis of Fmoc-Pro-Pro-OH ... 8

Peptide Characterization Data ... 23

$TAD1_{1-39}$ $NHNH_2$ pS6 ... 23
$TAD1_{1-39}$ $NHNH_2$ pS9 ... 24
$TAD1_{1-39}$ $NHNH_2$ pS15 ... 25
$TAD1_{1-39}$ $NHNH_2$ pT18 ... 26
$TAD1_{1-39}$ $NHNH_2$ pS20 ... 27
$TAD1_{1-39}$ $NHNH_2$ pS33 ... 28
$TAD1_{1-39}$ $NHNH_2$ pS37 ... 29
$TAD1_{1-39}$ $NHNH_2$ pS15pT18pS20 ... 30

Expression and purification of p53ΔN ... 31

Protein Expression ... 31
Inclusion Body Isolation ... 31
Cleavage of His_6-SUMO tag and purification ... 32

Synthesis of full-length phospho-p53 variants via Native Chemical Ligation ... 34

Methods for Native Chemical Ligation ... 34

Procedure 1 ... 34
Procedure 2 ... 34
Procedure 3 ... 35
Procedure 4 ... 36
Procedure 5 ... 36

Characterization data for full-length p53 variants ... 38

$p53_{1-393}$ pS6 (**4a**) ... 38
$p53_{1-393}$ pS9 (**4b**) ... 39
$p53_{1-393}$ Pra1,pS15 (**4c**) ... 40
$p53_{1-393}$ pT18 (**4d**) ... 41
$p53_{1-393}$ pS20 (**4e**) ... 42
$p53_{1-393}$ pS33 (**4f**) ... 43
$p53_{1-393}$ pS37 (**4g**) ... 44
$p53_{1-393}$ pS15pT18pS20 (**4h**) ... 45

Cross Validation of 'designer' phospho-p53 library and p53-specific antibodies by Western Blot ... 47

Materials and Methods ... 47

Quantitative Analysis of phos-p53 in Western Blot ... 49

Supplementary references ... 53

TAD1 Peptide Synthesis & Characterization

Materials & Methods

Chlorotrityl resin functionalization

Chlorotrityl chloride resin, 1 mmol (polystyrene, 1 % DVB, 100-200 mesh, Novabiochem® 1.0-1.8 mmol/g substitution or Anaspec® 0.6-1.4 mmol/g substitution) was swollen in 3 mL of DMF on ice with gentle agitation for 15 min. Then, 3 eq (3 mmol) of triethylamine were added, followed by drop-wise addition of hydrazine hydrate (2 mmol) dissolved in 1 mL of DMF, to the resin on ice under continuous stirring. The reaction was allowed to stir at room temperature for 1 hr, followed by addition of 2 mL of methanol used to cap any unreacted sites. Resin capping was conducted for 15 min, followed by filtering the resin and conducting the following batch washes for 1 min with nitrogen bubbling: 2 x 8 mL DMF, 2 x 5 mL H_2O, 2 x 5 mL DMF, 2 x 5 mL MeOH, 1 x 5 mL DMF. As chlorotrityl-hydrazine resin is known to be unstable, the first amino acid (Fmoc-Ala-OH) was loaded onto the resin. Generally, the Fmoc amino acid (4 eq = 4 mmol) was dissolved in DMF to a concentration of 0.5 M, to which 4 mmol of Oxyma Pure were added together with 4 eq (4 mmol) DIC. Activated amino acid was added immediately to the resin and let to react under nitrogen bubbling for 1h. Reaction was then filtered out under vacuum and resin was washed with DMF (1 min batch wash bubbling, 1 min continuous DMF flow covering the resin, 1 min batch bubbling wash) and the coupling reaction was repeated, with consecutive DMF wash, followed by DCM wash of the resin. Reaction progression was monitored with qualitative Keiser test (Anaspec®). The substituted resin was dried under vacuum overnight and resin substitution was assessed with quantitative Fmoc-release test the following day. For the resin substitution test 3 samples of approximately 5 mg of the resin were added to 3 separate Eppendorf tubes and covered with 1 mL of 20% piperidine and left to react for 10 min with continuous shaking. Then, the resin was let to sediment and 100 µL from top of the liquid were aspirated and diluted with 900 µL fresh 20% piperidine in DMF. UV absorbance at 290 nm of the diluted sample was measured in a quartz cuvette (1 cm) and resin loading was calculated with the equation:

$$\text{Loading (mmol/g)} = 10^4 \times \frac{V \times A_{290}}{\varepsilon \times d \times m_s}$$

where V= volume (L), d = path length (cm), ε = 5800 M^{-1} cm^{-1}, m_S – mass of the resin sample (g). The average of the three readings was taken to calculate the resin substitution and the value obtained was used for later calculations for peptide synthesis. Resin loading was determined to be between 0.35-0.50 mmol/g indicating 35-50% conversion. Resin was stored under vacuum until needed.

General Procedure for SPPS

TAD1 p53 peptide hydrazides (39-mer) were synthesized as described previously by Margiola *et al.* (2021).[1] Briefly, peptides were synthesized on 0.1 mmol scale on 2-chlorotrityl chloride solid support (Novabiochem®) as acyl hydrazides with Fmoc-protected amino acid building blocks. All reagents were purchased from Novabiochem® or Fluorochem®, unless states otherwise. The N-terminal amino acid in the sequence - methionine, was changed to its isostere - norleucine to avoid issues with oxidation. The unmodified peptides (no PTMs) coupling reactions were performed with the consecutive amino acid, Oxyma Pure and DIC (4 eq/4eq/4eq) for 1 hr at room temperature or at 75°C for 5 min. Coupling reactions were repeated twice (total number of couplings = 2) to ensure reaction completion. Increasing the coupling temperature and concomitantly decreasing the coupling time did not substantially improve the isolated yield nor the purity of the product, but served to speed up the synthetic workflow. Deprotections of the Fmoc group were performed twice with 20% piperidine in DMF (2 min and 8 min). Phosphoserine and phosphothreonine were incorporated using mono-protected building block Fmoc-Ser(PO(OBzl)OH)-OH, and Fmoc-Thr(PO(OBzl)OH)-OH. After coupling of a phosphoserine/ phosphothreonine, conditions were modified to couplings with amino acid, Oxyma Pure, DIC and DIPEA (5 eq/5 eq/5 eq/2 eq) and deprotection cycles were conducted twice with 5% piperazine in DMF (3 and 10 min). Synthesis was automated with a Biotage® Initiator+ Alstra™ peptide synthesizer, with manual couplings performed for loading of the first amino acid and coupling of the phosphoserine/ phosphothreonine. The efficiency of amino acid loading was monitored with Keiser’s test.

Table S1. Coupling conditions for TAD1 phosphopeptide hydrazide synthesis.

Peptide	Coupling Conditions	Special Amino Acids
**pS6**	RT, 1 hr	
**pS9**	RT, 1 hr	
**pS15**	75°C, 5 min; RT, 60 min for pAA and 1st AA after pAA	Fmoc-Pro-Pro-OH, Fmoc-propargylglycine
**pT18**	RT, 1 hr	
**pS20**	RT, 1 hr	
**pS33**	75°C, 5 min; RT, 60 min for pAA and 1st AA after pAA	Fmoc-Pro-Pro-OH
**pS37**	75°C, 5 min; RT, 60 min for pAA and 1st AA after pAA	
**pS15pT18pS20**	RT, 1 hr, extra equivalent of activated amino acid ester after each phosphoamino acid addition	

Peptide Cleavage and Purification

Following the synthesis, peptides were cleaved off the resin with 10 mL/0.1 mmol resin of reagent K (82.5% TFA, 5% thioanisole, 5% H_2O, 5% phenol, 2.5% 1,2-ethanedithiol). After 2 hrs the cleavage cocktail was filtered, and resin was washed with additional TFA (1 V, 10 mL/0.1 mmol resin) which was combined with the filtrate. TFA was evaporated under a stream of nitrogen and crude peptide was precipitated and washed thrice with ice-cold diethyl ether. The crude precipitate was dissolved in a minimal volume of 50% MeCN (0.1% TFA) and lyophilized.

The lyophilized crude material was dissolved in 25% MeCN (0.1% TFA), filtered and purified on an Agilent Infinity II Preparative System using a gradient of water: acetonitrile (0.1% TFA) on a C18 preparative column (Zorbax 300BS-C18 21.2x150 mm, 7 µm particle size) and collected in 6 mL fractions. Aliquots of collected fractions were analyzed on RP-HPLC and HRMS-ESI for assessment of purity. Fractions containing product were combined and lyophilized for long-term storage.

Analytical HPLC

Samples of peptides and proteins were run on analytical RP-HPLC (Agilent 1260 Infinity II) on a C3 column for proteins (300SB- Zorbax SB-C3, 5 µm particle size, 4.6 x 150 mm) and C18 for peptides with a water: acetonitrile gradient. HPLC buffers were acidified by the addition of 0.1% TFA. The absorbance was recorded at 214 nm and 280 nm. Integrated peaks at 214 nm of absorbance were used for quantification of the analytes with a calculated extinction coefficient.[2]

ESI-HRMS of peptides and proteins

Lyophilized samples of peptides and proteins were dissolved in a minimal volume of 50% acetonitrile (0.1% formic acid) and diluted to 1 µM. 1-2 µl of each sample were injected onto an Acquity I UPLC (Waters®) and resolved on a C4 column (300 Å, 1.7 µm, 2.1 mm x 50 mm) at 40 °C with a gradient of 5-75% water: acetonitrile acidified with 0.1% formic acid, without measuring the UV absorbance of the analyte. The analyte coming out of the column was directed to the MS-ESI (Xevo G2-XS QToF, Waters®) for mass detection and analysis. Data was collected at 50-3000 Da for peptides and 500-3000 Da for proteins. The ESI- HRMS was run in positive mode with the following parameters: source capillary voltage 3.01 kV, source temperature 120 °C, sampling cone voltage 60 V, source offset voltage 50 V, desolvation temperature 350 °C, cone gas flow 10 L/hr, desolvation gas flow 600 L/hr). Recorded protein mass spectra were deconvoluted with the MaxEnt1 algorithm.[3] The settings for deconvolution were: selection peaks lying between 500 and 1800 Da, assuming a Gaussian peak distribution with a half-peak width determined to be 0.35 Da and a resolution of 1.0 Da.

Reverse Phase purification of peptides and proteins

Peptides and proteins were purified on an Agilent Infinity II Preparative System with manual injections of 10-20 mL of the sample. The column used was Zorbax 300SB C3, 7 µm particle size, 21.2 x 150 mm and the gradient of water: acetonitrile (0.1% TFA). All HPLC buffers were degassed prior to the purification. Fractions were collected manually above a threshold of 200 mAu at A_{214}. Collected fractions were analyzed on analytical RP-HPLC and MS-ESI for identity and purity assessment. Pure fractions were pooled and lyophilized for long-term storage.

Gradient A: 20-60%B over 20 min at 1 mL/min for analytical runs, 4 mL/min for semi-preparative runs and 20 mL/min for preparative runs, RT

Gradient B: 25-55%B over 40 min at 1 mL/min for analytical runs, 4 mL/min for semi-preparative runs and 20 mL/min for preparative runs, RT

Gradient C: 25-45%B over 20 min at 1 mL/min for analytical runs, 4 mL/min for semi-preparative runs and 20 mL/min for preparative runs, RT

Gradient D: 30-55%B over 45 min at 1 mL/min for analytical runs, 4 mL/min for semi-preparative runs and 20 mL/min for preparative runs, RT

Truncation -Pro as a challenge in purifications

During the synthesis of TAD1 peptide hydrazides we observed a persistent -Pro truncation of the peptide. Removing this impurity required multiple rounds of purification negatively affecting yields. This truncation was likely caused by the P12-P13 sequence. To overcome this, a diproline building block (Fmoc-Pro-Pro-OH) was synthesized and purified as detailed below. NMR and the MS spectrum of the final product are presented in Figures S5-6. Fmoc-Pro-Pro-OH was used in synthesis of TAD1 pS15 and TAD1 pS33 (see analytical data in Figures S8 and S10, respectively), successfully eliminating the truncation. This strategy improved crude purity and reduced purification efforts, see Figure S1 for comparison of crude peptide purity by ESI-HRMS.

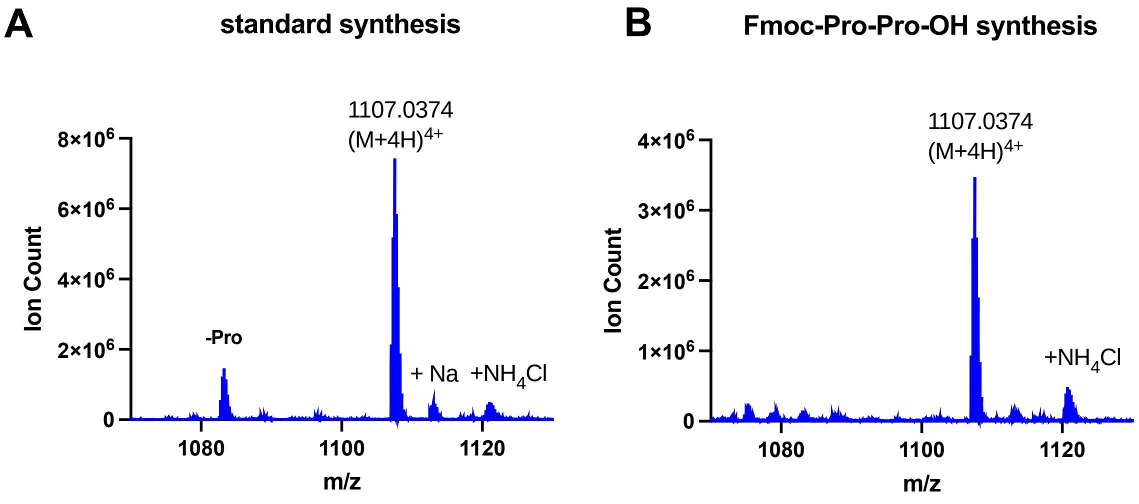

Figure S1. Fmoc-Pro-Pro-OH improves synthesis of p53 TAD1. (A) ESI-HRMS of crude TAD1 peptide, following the 'standard synthesis' of consecutive couplings of prolines. (B) ESI-HRMS of crude TAD1 peptide using Fmoc-Pro-Pro-OH.

Synthesis of Fmoc-Pro-Pro-OH

Solution-based synthesis of Fmoc-Pro-Pro-OH was based on a protocol described by Minkovich and others.[4]

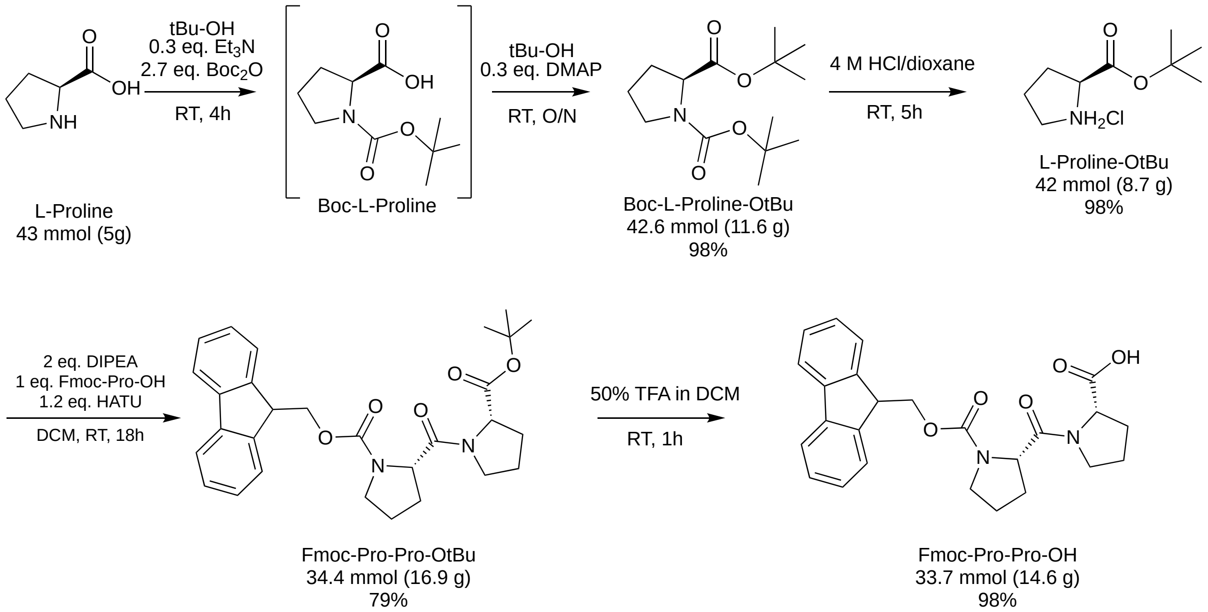

Scheme S1. Synthesis of Fmoc-Pro-Pro-OH.

Boc-Pro-OtBu

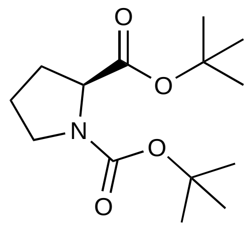

Proline tert-butyl ester was prepared in the following fashion. L-proline (5 g, 43.4 mmol) was dissolved in 75 mL of tert-butanol in a round-bottom flask, 0.3 eq of triethylamine was added (13 mmol, 1.82 mL) followed by the addition of 2.7 eq. of ditertbutyl dicarbonate (117.2 mmol, 26.9 mL) and the reaction was allowed to proceed for 4 hr at room temperature with continuous stirring (Boc-protection). Then, 0.3 eq. of dimethylaminopyridine (DMAP) was added (13 mmol, 1.6 g) and the reaction was stirred overnight at RT (carboxylic acid protection with tert-butyl). Reaction completion was assessed with TLC and NMR, with quantitative conversion to the desired product. Upon reaction completion, tert-butanol was removed under vacuum and the remainder

dissolved in 2 vol (160 mL) of ethyl acetate, which was washed with 1 vol (80 mL) of 0.1 M HCl, 1 vol. (80 mL) saturated bicarbonate, 1 vol. brine. The organic layer was dried over anhydrous magnesium sulfate, and the organic solvent was removed under vacuum. Boc-Pro-OtBu was isolated in 98% yield (11.6 g, 42.6 mmol).

^{1}H NMR (400 MHz, Chloroform-d) δ 4.07 (ddd, J = 29.7, 8.7, 3.3 Hz, 1H), 3.53 – 3.24 (m, 2H), 2.21 – 2.00 (m, 1H), 1.92 – 1.67 (m, 3H), 1.41 – 1.33 (m, 18H). The peak at δ 7.30 corresponds to $CDCl_3$. **^{13}C NMR** (101 MHz, Chloroform-*d*) δ 172.10, 154.19, 80.64, 79.23, 59.57, 46.40, 29.78, 28.25, 27.89, 24.11. Singlet peak at δ 76.80 is $CDCl_3$.

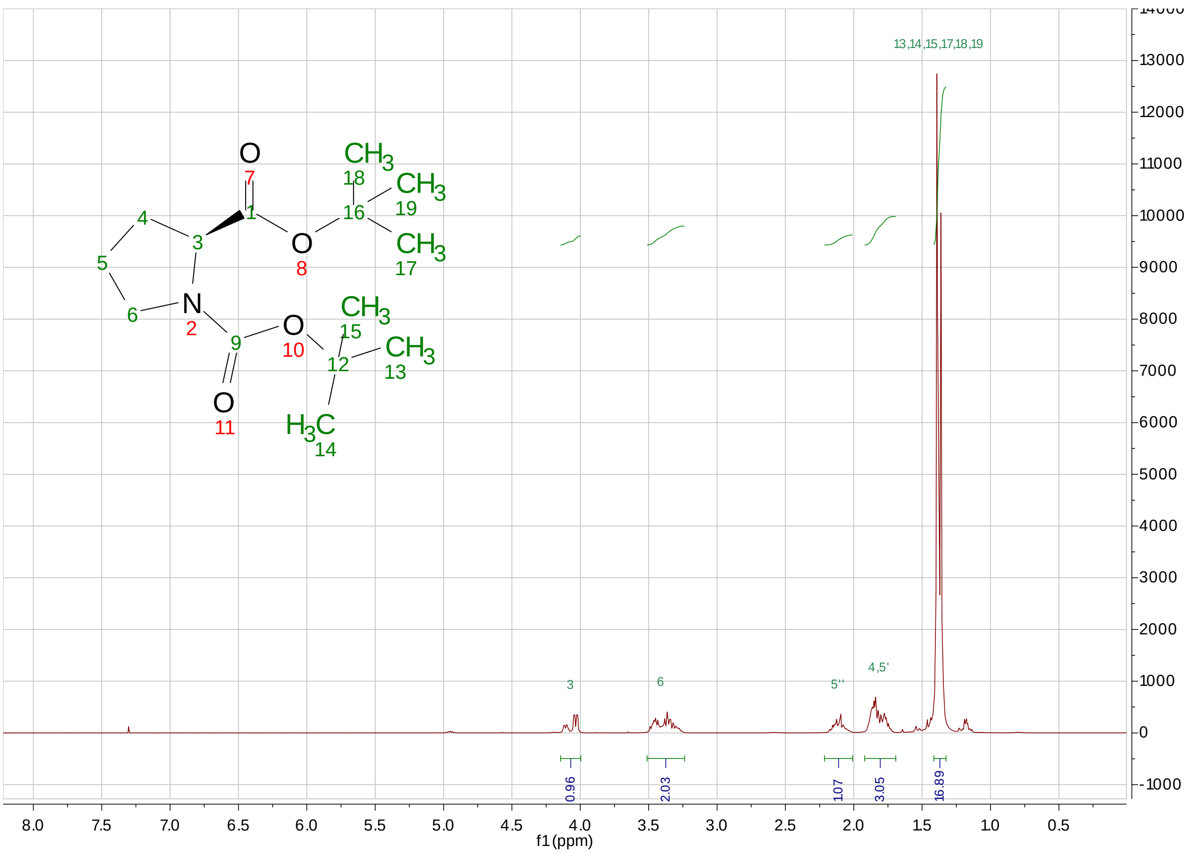

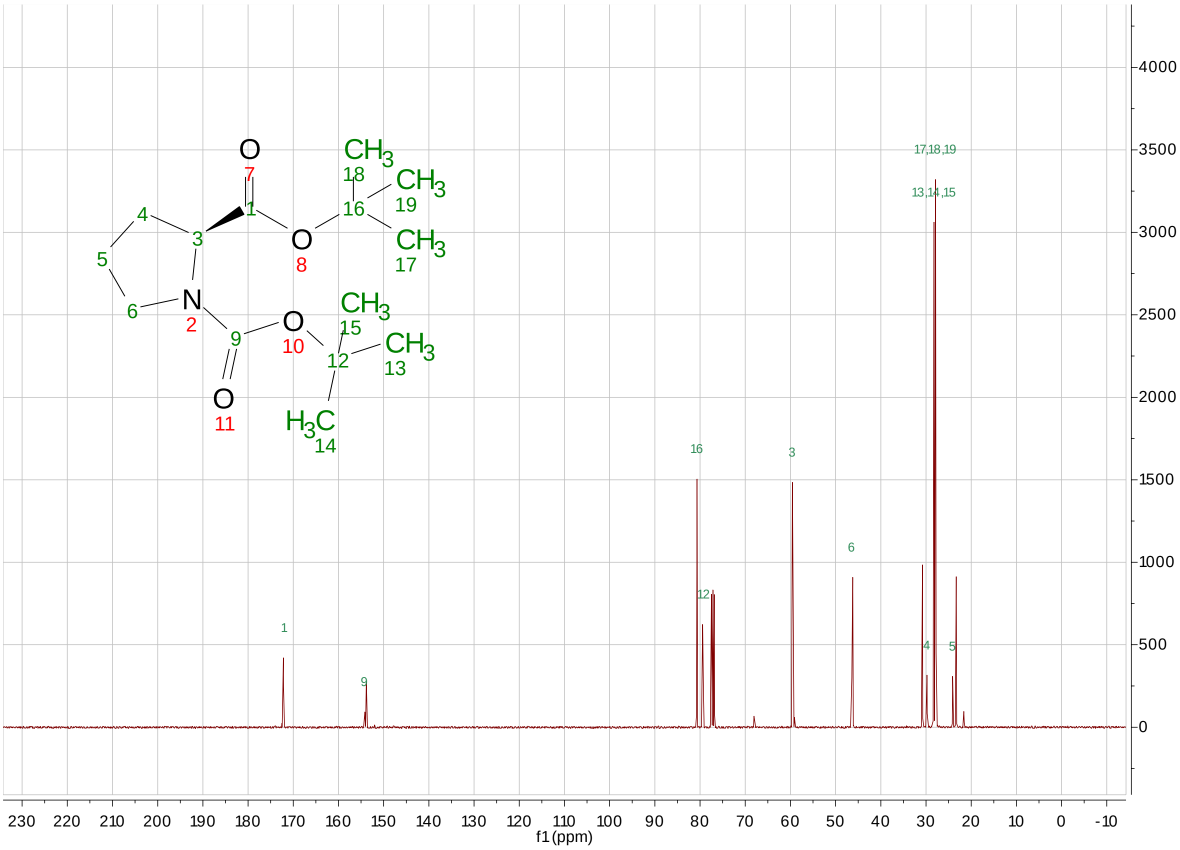

Figure S2. ^{1}H NMR (top) and ^{13}C NMR (bottom) spectra of Boc-L-Pro-OtBu.

N-Pro-OtBu

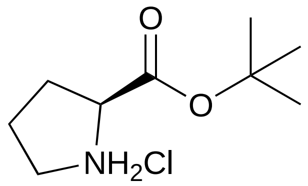

11.6 g (42.6 mmol) of Boc-L-Pro-OtBu were treated with 4 M HCl in dioxane (12 mL) for 2h to remove the N-Boc protecting group. Reaction progress was followed by TLC (10% methanol in DCM). Upon completion, volatiles were removed under vacuum and crude reaction mixture was directly used in the next step (42 mmol isolated as hydrochloride salt, 8.7 g).

^{1}H NMR (400 MHz, Chloroform-*d*) δ 10.49 – 10.40 (m, 1H), 4.52 – 3.98 (m, 1H), 3.59 – 3.38 (m, 2H), 2.41 – 2.28 (m, 1H), 2.24 – 2.02 (m, 2H), 2.02 – 1.90 (m, 1H), 1.83 (dddt, *J* = 20.8, 14.3, 7.6, 3.7 Hz, 1H), 1.44 (s, 9H). Singlet peak at δ 3.64 is residual dioxane. Singlet peak at δ 7.40 is $CDCl_3$.

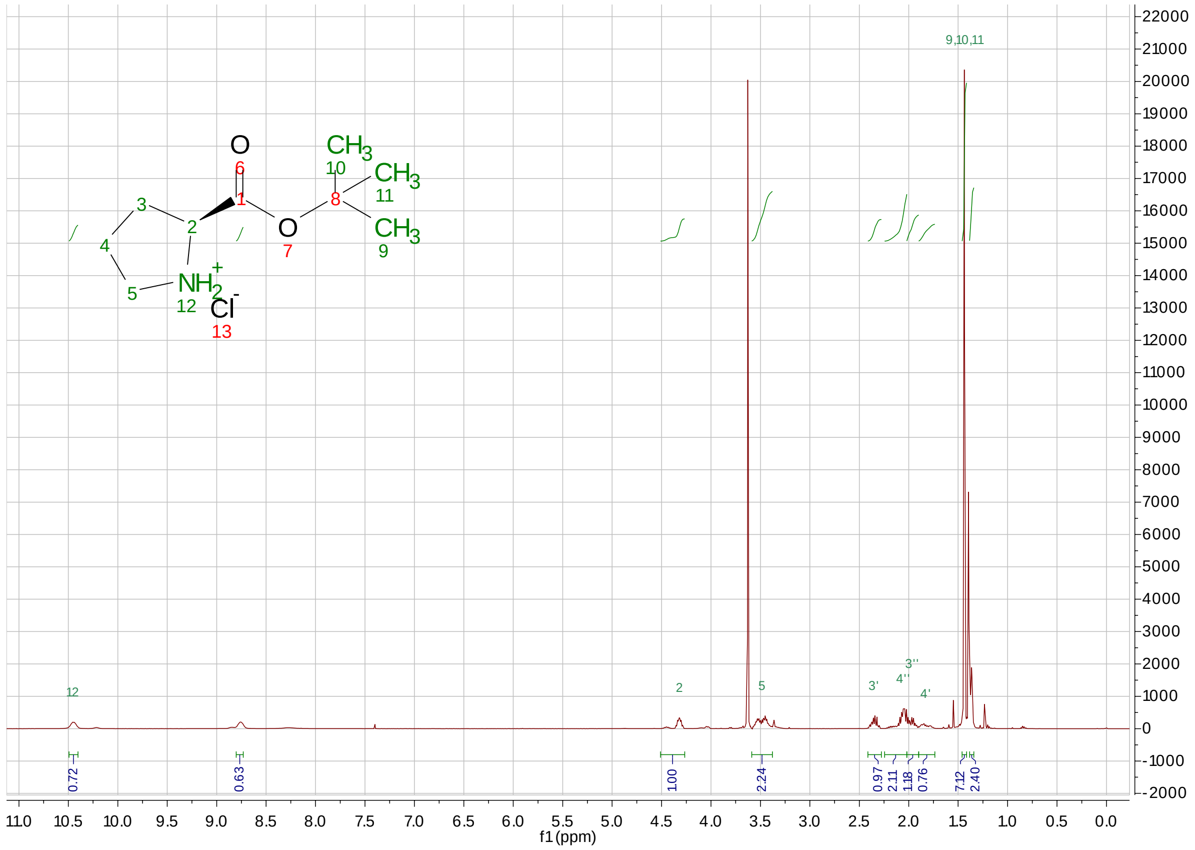

Figure S3. Characterisation of Pro-OtBu by NMR spectroscopy. ^{1}H NMR (top) and ^{13}C NMR (bottom)

Fmoc-Pro-Pro-OtBu

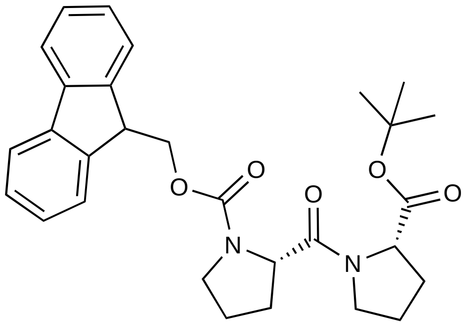

Crude Pro-OtBu 8.7 g (42 mmol) was dissolved in 80 mL of DCM, 2 eq. of diisopropylethylamine (DIPEA) was added (15.6 mL), with 1 eq. of Fmoc-Pro-OH (42 mmol, 14.4 g) and 1.2 eq of HATU (19.4 g). The reaction was stirred for 18 h at room temperature. Following the overnight reaction, DCM was removed under vacuum and the remainder was dissolved in 80 mL of ethyl acetate, washed once with 1 M HCl, washed thrice with water, once with brine, and dried over magnesium sulfate. Ethyl acetate was removed under vacuum and the crude was isolated.

The crude was then split into 5-8 g aliquots and dry-loaded onto silica gel and purified with a step-wise gradient of 0-100% of ethyl acetate in hexane, with product elution at 50% ethyl acetate in hexane. Fractions containing pure Fmoc-Pro-Pro-OtBu were combined and solvents evaporated. The isolated product presented as a crystalline solid (16.9 g, 34.4 mmol, 79% isolated yield).

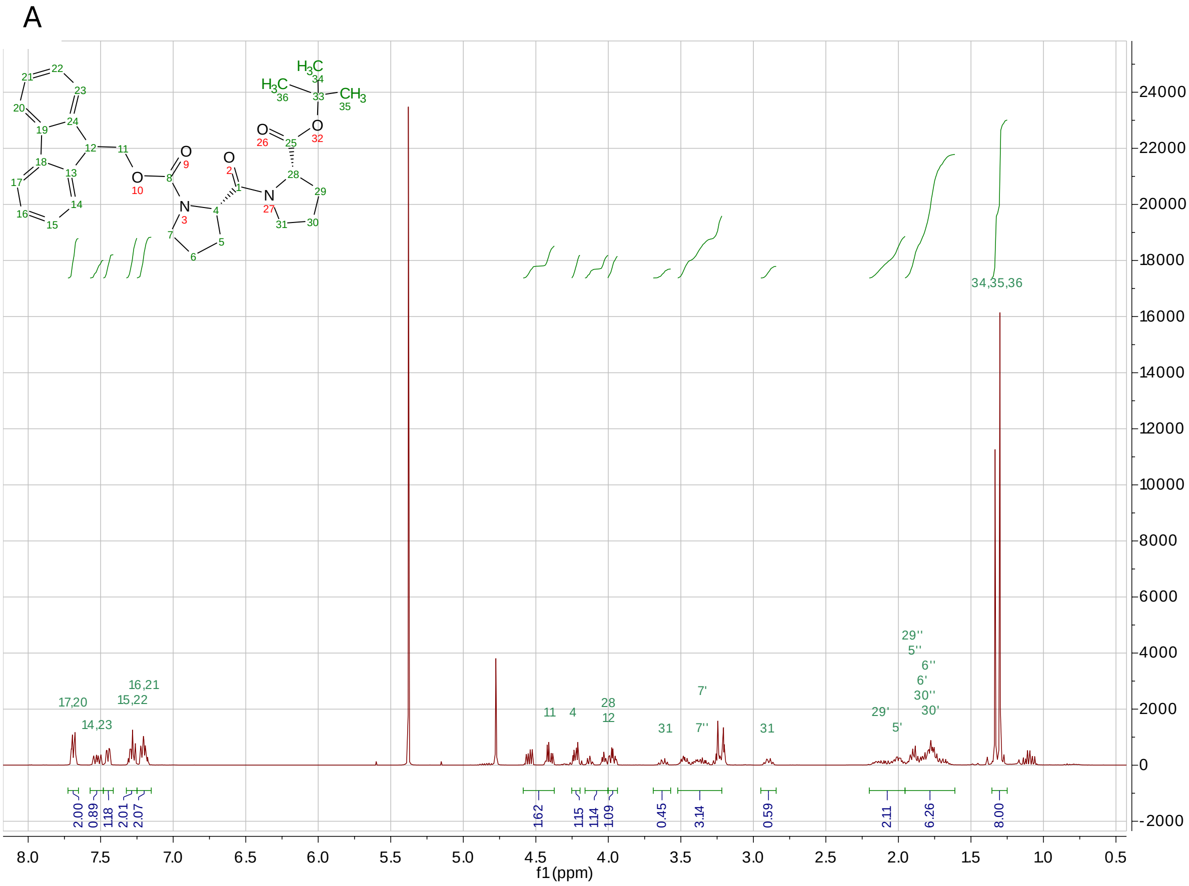

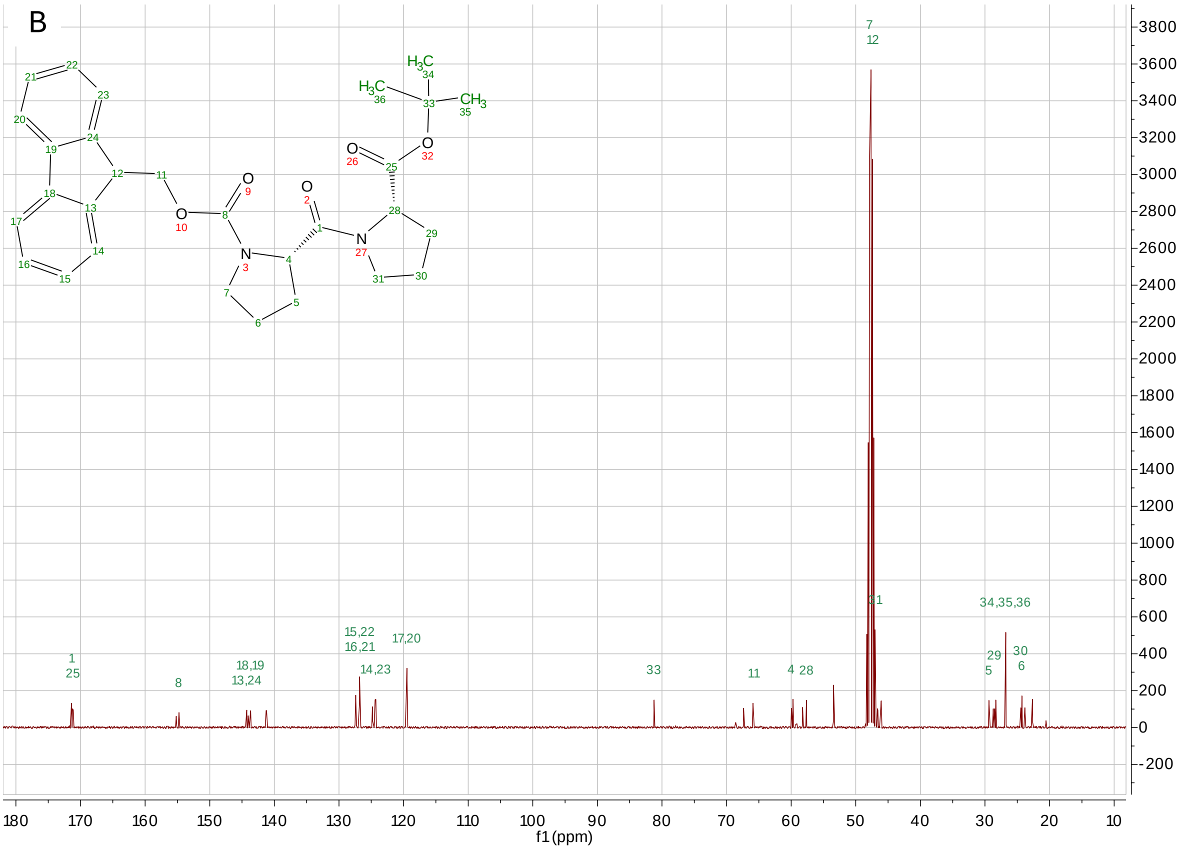

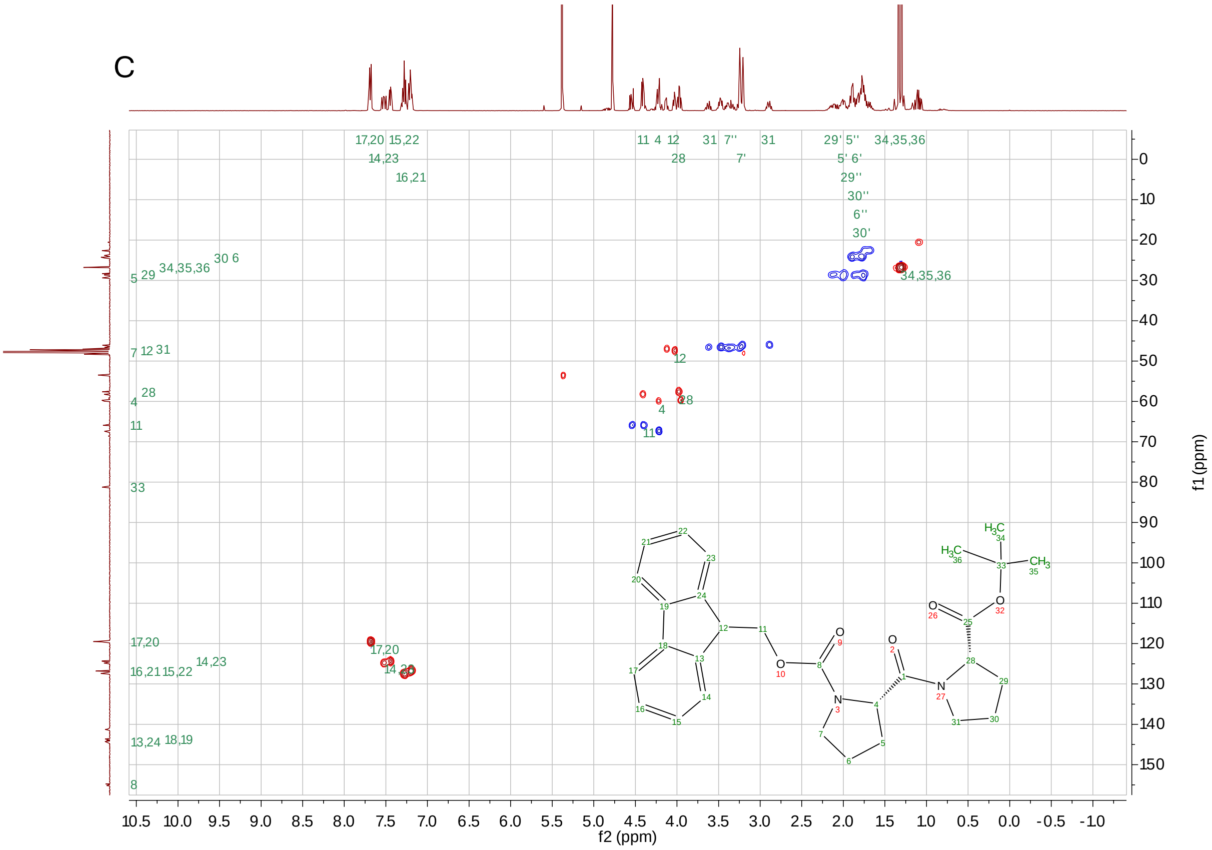

Figure S4. Characterisation of Fmoc-Pro-Pro-OtBu by NMR spectroscopy. ^{1}H NMR (A) and ^{13}C NMR (B), HSQC (C).

^{1}H NMR (400 MHz, Methanol-d_4) δ 7.69 (ddd, J = 7.6, 2.2, 1.1 Hz, 2H), 7.52 (ddd, J = 12.0, 7.5, 1.0 Hz, 1H), 7.48 – 7.41 (m, 1H), 7.32 – 7.24 (m, 2H), 7.24 – 7.15 (m, 2H), 4.60 – 4.50 (m, 1H), 4.46 – 4.36 (m, 1H), 4.35 – 4.20 (m, 1H), 4.24 – 4.09 (m, 1H), 4.07 – 3.92 (m, 2H), 3.62 (dt, J = 9.9, 7.0 Hz, 0H), 3.53 – 3.17 (m, 3H), 2.95 – 2.84 (m, 1H), 2.20 – 1.93 (m, 1H), 1.89 (s, 1H), 1.96 – 1.61 (m, 4H), 1.32 (d, J = 13.3 Hz, 9H). Singlet peak at δ 5.37 is residual dichloromethane, singlet peak at δ 4.77 is HDO, multiplet peak at δ 3.20 is MeOD.

^{13}C NMR (101 MHz, Methanol-d_4) δ 171.40, 171.24, 154.77, 144.27, 143.66, 126.82, 126.80, 124.29, 119.50, 81.22, 67.38, 59.95, 58.24, 53.45, 48.29, 47.01, 28.50, 28.32, 26.79, 24.46, 24.28. Peak at δ 53.35 is residual dichloromethane. Peak at δ 48.90 is MeOD.

Fmoc-Pro-Pro-OH

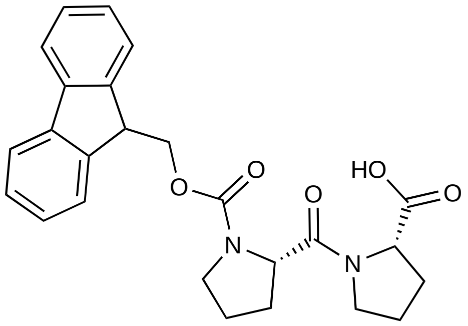

Fmoc-Pro-Pro-OtBu (16.9 g, 34.4 mmol) was dissolved in 80 mL of 50% TFA in DCM to remove the tert-butyl ester, which was quantitative. Upon completion, the reaction was diluted with 2 volumes of toluene (known TFA azeotrope) and volatiles were removed under vacuum. The product was then washed twice with toluene with solvent removal under vacuum. The isolated product (98% yield, 14.6 g) was then lyophilized thrice to remove any remaining TFA. It presented either as a light yellow-oil (just after lyophilization) or white to light-yellow powder (following solvent removal under vacuum). Compound identity was confirmed by ESI-MS (expected mass $(M+H)^+$ 435.1914, detected mass $(M+H)^+$ 435.1919 Da).

VT NMR Method

Variable temperature (VT) NMR experiments were performed using a Bruker Ascend 400 MHz spectrometer equipped with a room temperature PI HR-BBO400S1-BBF/H/D-5.0-Z SP iProbe. 1D 1H (zg30) and 1H -13C HSQC (hsqcedetgp) experiments were acquired at 20 K increments from 293 K to 393 K, at which point coalescence of Fmoc-Pro-Pro-OH signals had clearly occurred. 1D 1H experiments were performed using 16 scans (ns) with a spectral window of 12.5 ppm to -0.5 ppm. 2D 1H -13C HSQC experiments were performed using 4 scans (ns), 8 dummy scans (ds) and a coupling constant of 145 Hz (cnst2). The spectral window for the 1H -13C HSQC experiment was 12.5 ppm to -0.5 ppm for the 1H (F2) axes and 210 ppm to -10 ppm for the 13C (F1) axes. In the F2 and F1 frequency axes, 2048 and 256 data points were collected, respectively. Once acquired, all spectra were then processed using TopSpin 3.6.5 and exported to MNova 14.3.1-31739 for final interpretation.

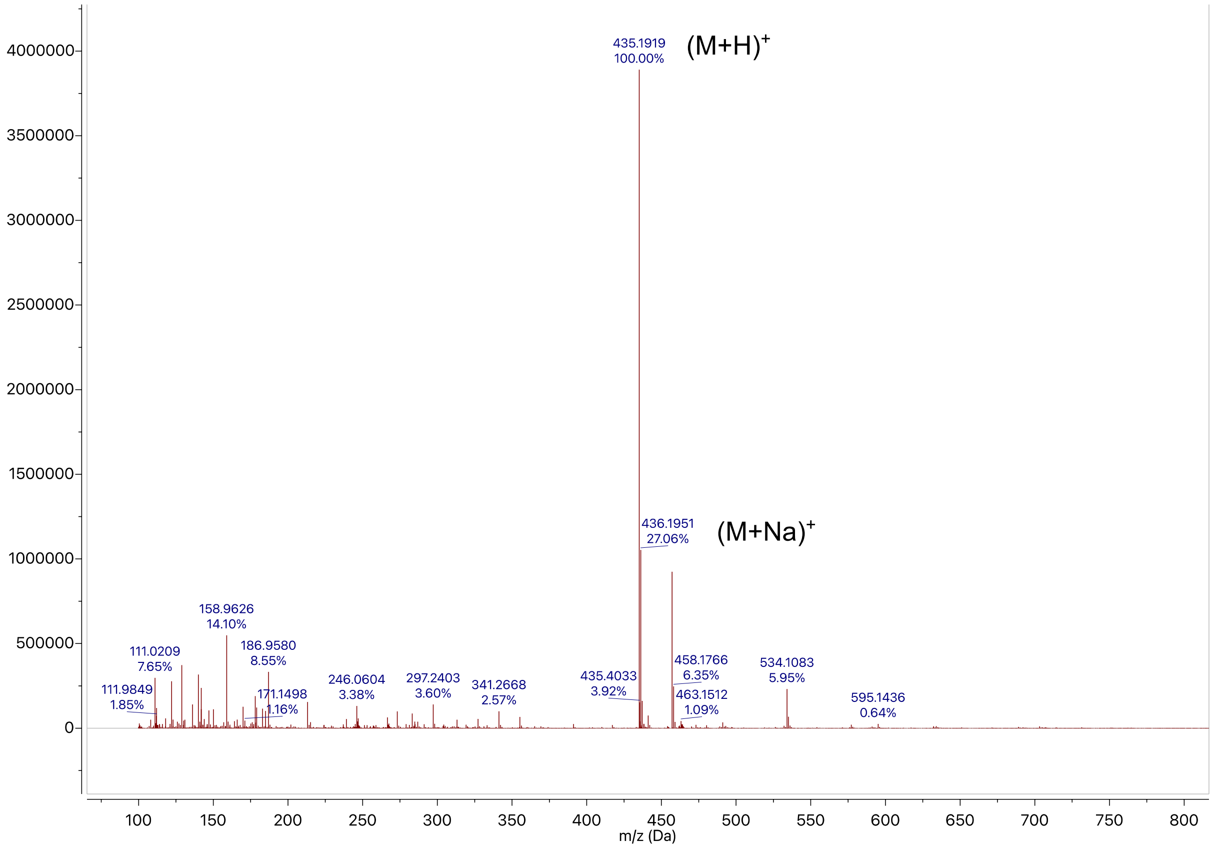

Figure S5. ESI-HRMS of Fmoc-Pro-Pro-OH, expected mass $(M+H)^+$ 435.1914, detected mass $(M+H)^+$ 435.1919 Da.

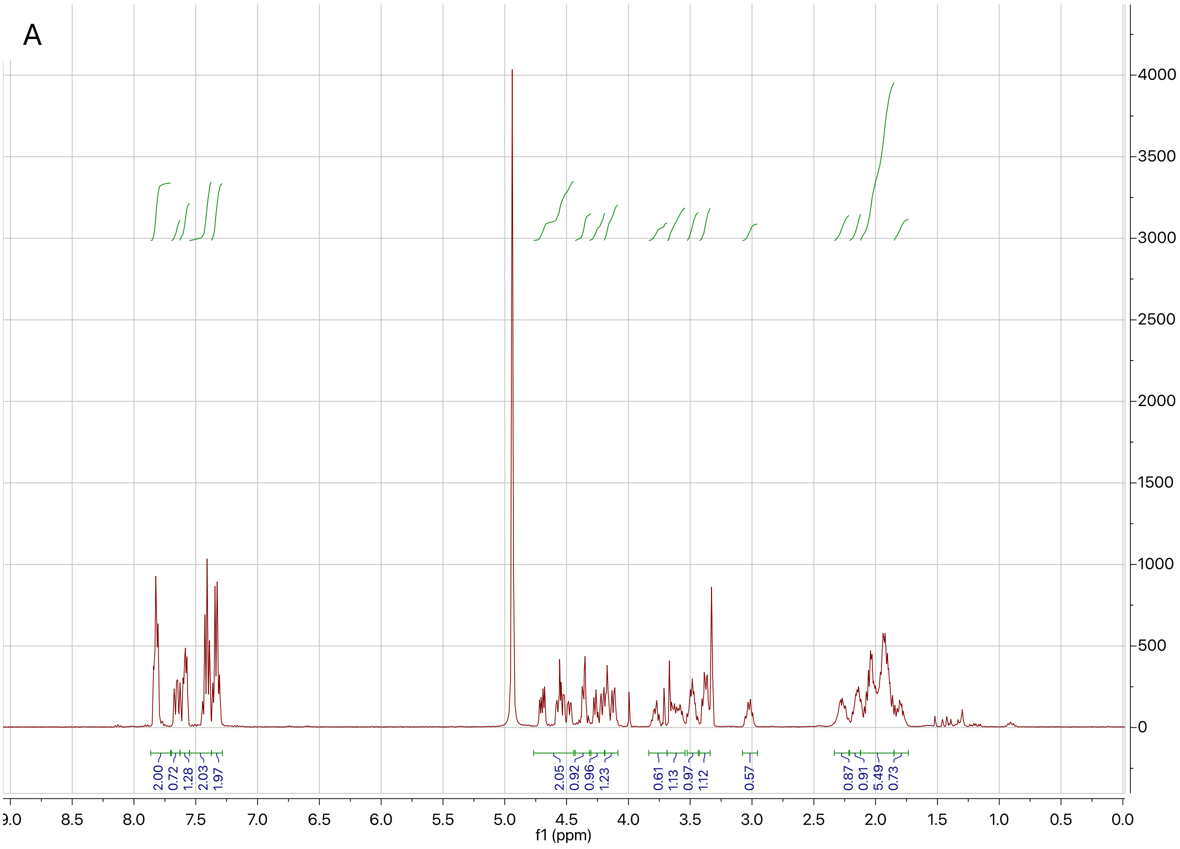
A
2.00
0.72
1.28
2.03
1.97
2.05
0.92
0.96
1.23
0.61
1.13
0.97
1.12
0.57
0.87
0.91
5.49
0.73
f1 (ppm)

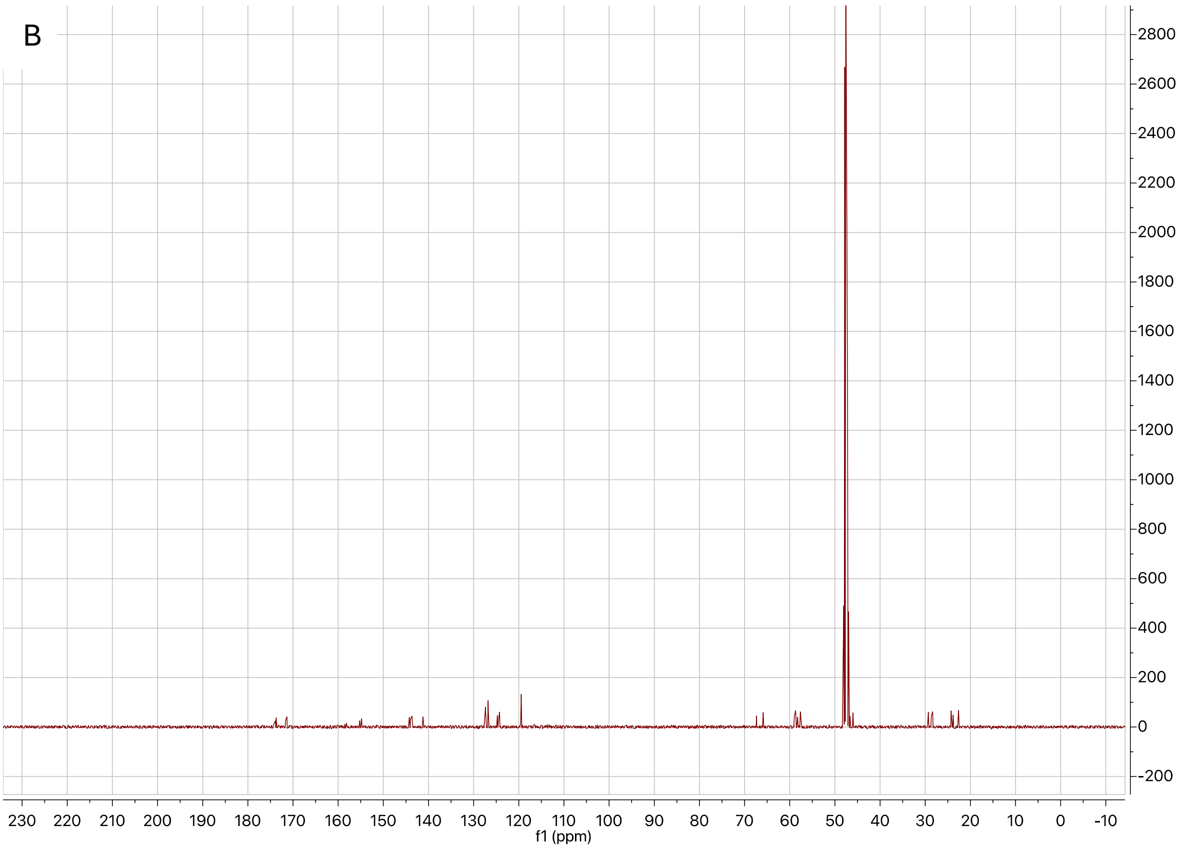
B
f1 (ppm)

C

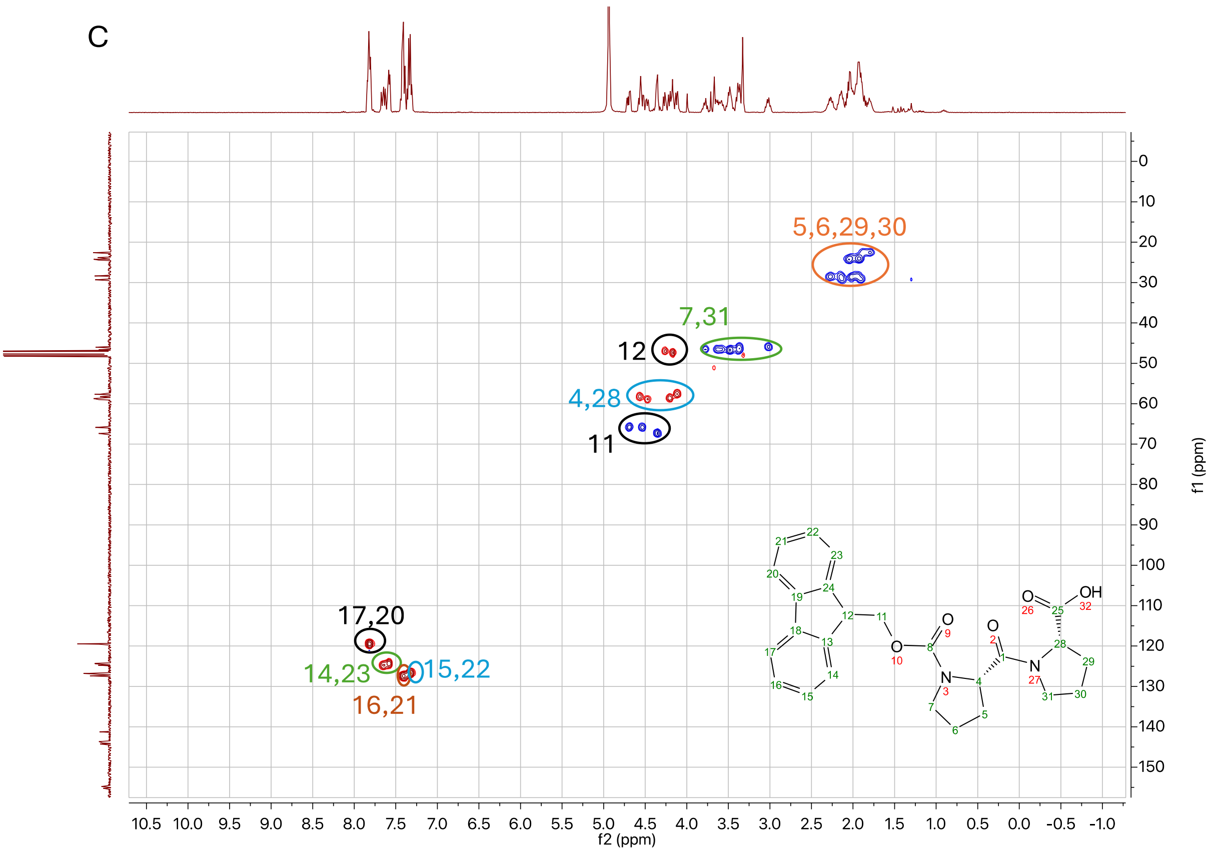
5,6,29,30
7,31
12
4,28
11
17,20
14,23
15,22
16,21
f2 (ppm)
f1 (ppm)

D

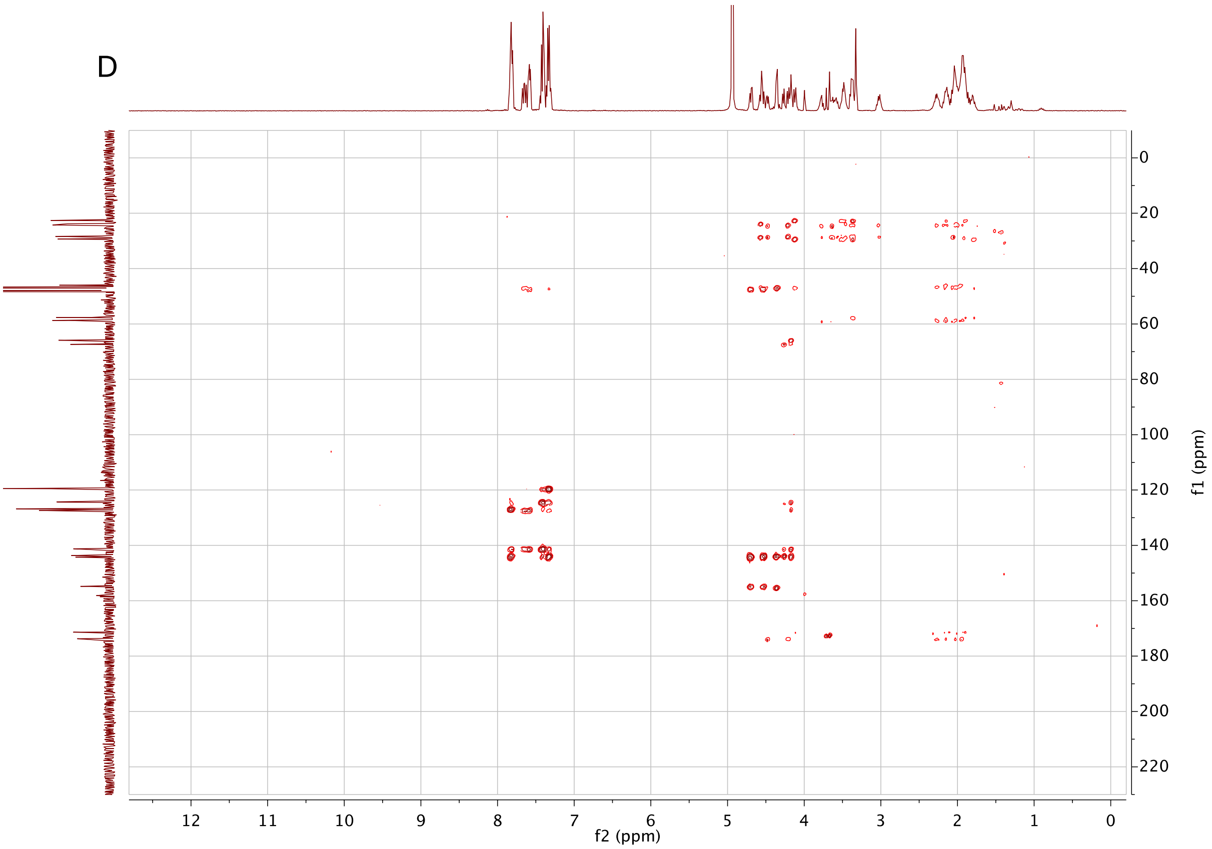
f2 (ppm)
f1 (ppm)

Figure S6. Characterisation of Fmoc-Pro-Pro-OH by NMR Spectroscopy. ^{1}H NMR (A) and ^{13}C NMR (B), HSQC (C), HMBC (D).

^{1}H NMR (400 MHz, Methanol-d_4) δ 7.86 – 7.70 (m, 2H), 7.70 – 7.63 (m, 1H), 7.63 – 7.55 (m, 1H), 7.42 (q, *J* = 7.3 Hz, 2H), 7.34 (dt, *J* = 8.6, 6.6 Hz, 2H), 4.70 (ddd, *J* = 10.9, 5.1, 1.5 Hz, 1H), 4.62 – 4.55 (m, 1H), 4.55 – 4.40 (m, 1H), 4.36 (dt, *J* = 6.6, 2.3 Hz, 1H), 4.34 – 4.20 (m, 1H), 4.20 – 4.07 (m, 1H), 3.83 – 3.69 (m, 1H), 3.69 – 3.54 (m, 1H), 3.52 – 3.44 (m, 1H), 3.38 (dt, *J* = 7.3, 4.8 Hz, 1H), 3.03 (dt, *J* = 10.2, 6.9 Hz, 1H), 2.33 – 2.22 (m, 1H), 2.21 – 2.12 (m, 1H), 2.12 – 1.85 (m, 5H), 1.85 – 1.74 (m, 1H). Singlet at δ 4.95 is residual HDO, multiplet at δ 3.35 is MeOD.

^{13}C NMR (101 MHz, Methanol-d_4) δ 141.28, 127.43, 126.79, 119.55, 119.47, 67.36, 58.25, 57.66, 48.24, 47.18, 46.97, 28.65, 28.55, 28.36, 24.25. Peak at δ 48.90 is MeOD.

A

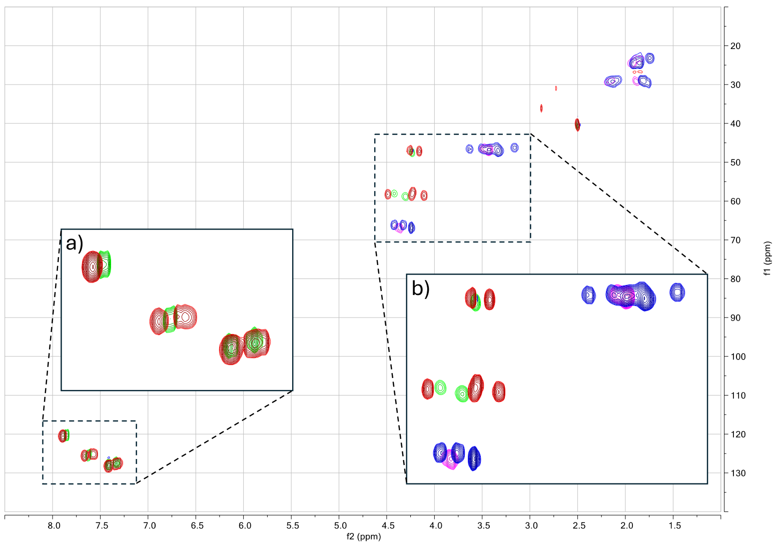

B

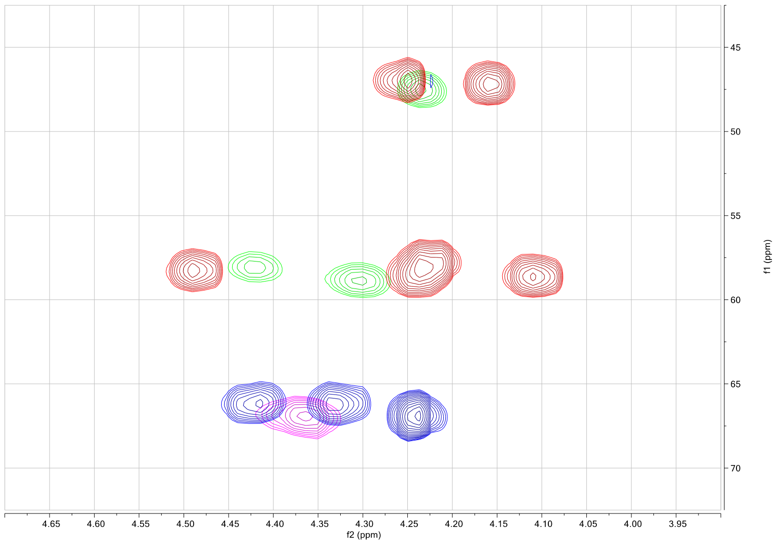

C

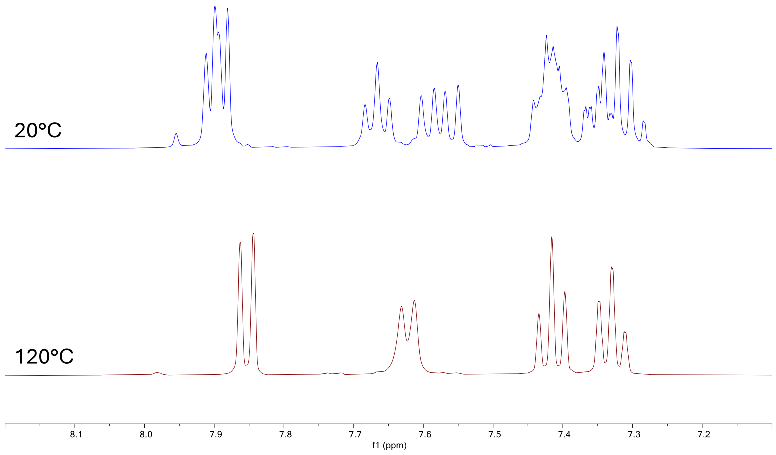

D

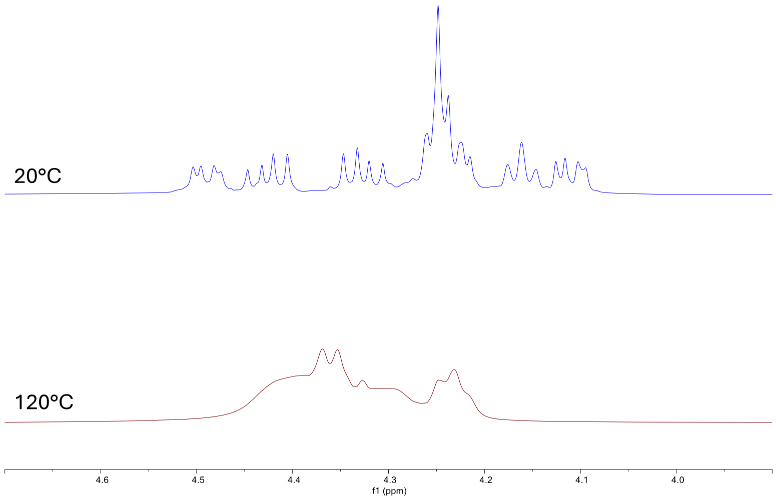

Figure S7. Temperature controlled HSQC 2D NMR of Fmoc-Pro-Pro-OH in d-DMSO. (**A)** Overlay of the 20 °C (red, positive and blue, negative) and 120 °C (green, positive and magenta, negative) 2D 1H-13C HSQC (hsqcedetgp) spectra for Fmoc-Pro-Pro-OH. Highlighted in a) and b) is the coalescence of the slow exchange peaks to individual intermediate-fast exchange peaks for the aromatic and α-proton region, respectively. This indicates that the presentation of Fmoc-Pro-Pro-OH as multiple peaks is related to conformational exchange and not the presence of secondary structural isomers or impurities. **(B)** Overlay of the 20 °C

(red, positive and blue, negative) and 120 °C (green, positive and magenta, negative) 2D 1H-13C HSQC (hsqcedetgp) spectra for Fmoc-Pro-Pro-OH showing the α-proton region from 3.9 ppm to 4.7 ppm in the 1H dimension and from 40 ppm to 75 ppm in the 13C dimension. **(C)** Stacked plot of the 20 °C (Blue) and 120 °C (Red) 1D 1H (zg30) spectra for Fmoc-Pro-Pro-OH showing the aromatic region from 7.1 ppm to 8.2 ppm. From 20 °C to 120 °C signals can be seen to coalesce from a complex set of slow exchange signals to the expected splitting pattern for the Fmoc group. **(D)** Stacked plot of the 20 °C (Blue) and 120 °C (Red) 1D 1H (zg30) spectra for Fmoc-Pro-Pro-OH showing the α-proton region from 3.9 ppm to 4.7 ppm. From 20 °C to 120 °C signals can be seen to coalesce from a complex set of overlapping slow exchange signals to broadened intermediate and intermediate-fast exchange signals. To improve clarity the 120 °C spectrum has been scaled by a factor of two relative to the 20 °C spectrum.

Peptide Characterization Data

TAD1$_{1-39}$ NHNH$_2$ pS6

Synthesised on a 0.129 mmol scale, 2.0% isolated yield, 11.4 mg. See Table S1 for synthetic adjustments.

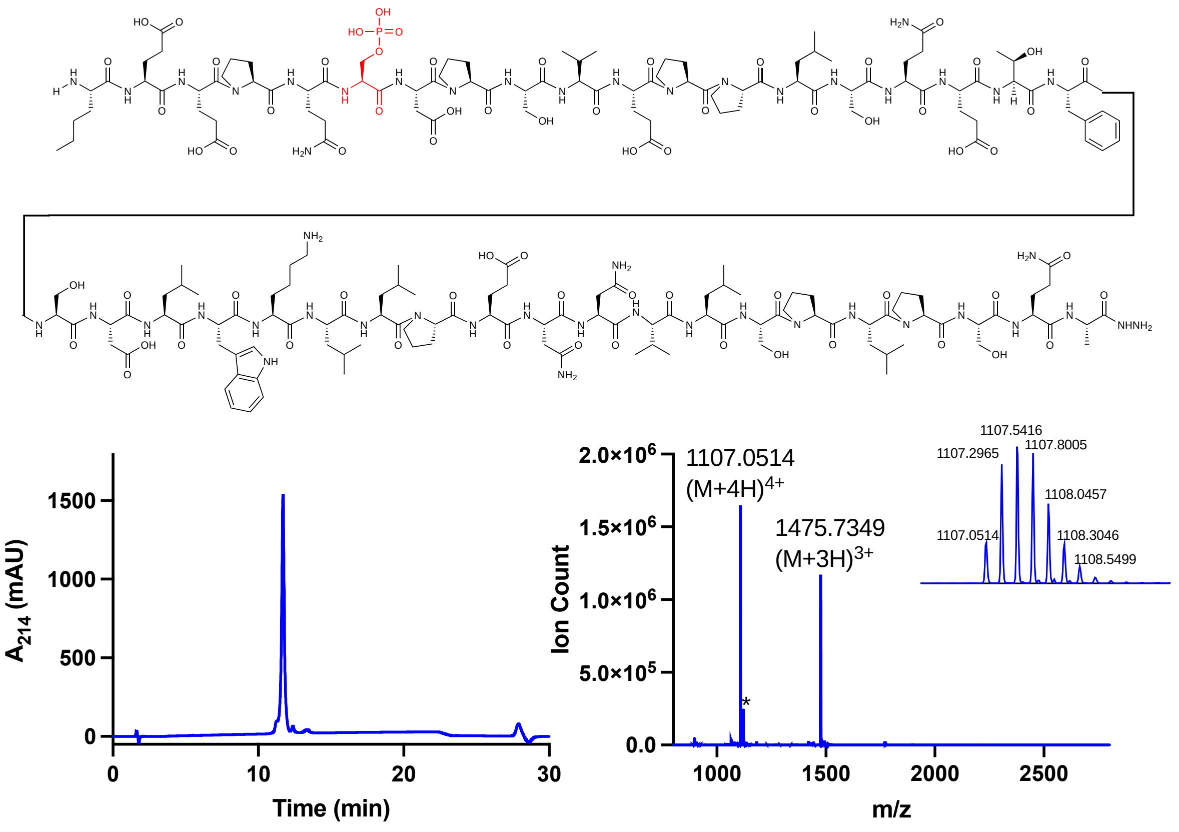

Figure S8. Characterization of TAD1$_{1-39}$ pS6 acyl hydrazide peptide (precursor to **1a**) by RP-HPLC (left) and ESI-HRMS (right). HPLC purity >90%, t_R= 11.7 min with gradient A, predicted mass: 4424.1465 Da, observed mass: 4424.1765 Da, peak denoted with an asterisk (*) is ammonium chloride adduct.

TAD1$_{1-39}$ NHNH$_2$ pS9

Synthesised on a 0.113 mmol scale, 1.7% isolated yield, 8.5 mg, see Table S1 for adjustments of the synthetic procedure.

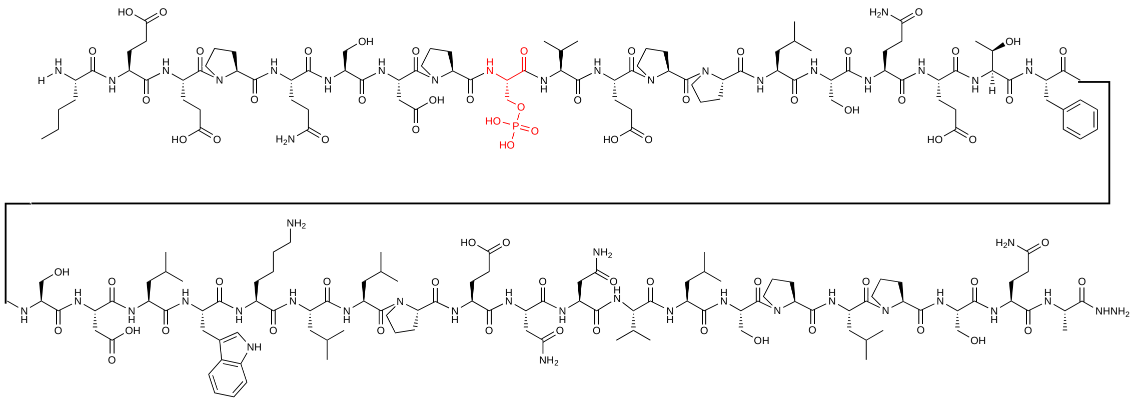

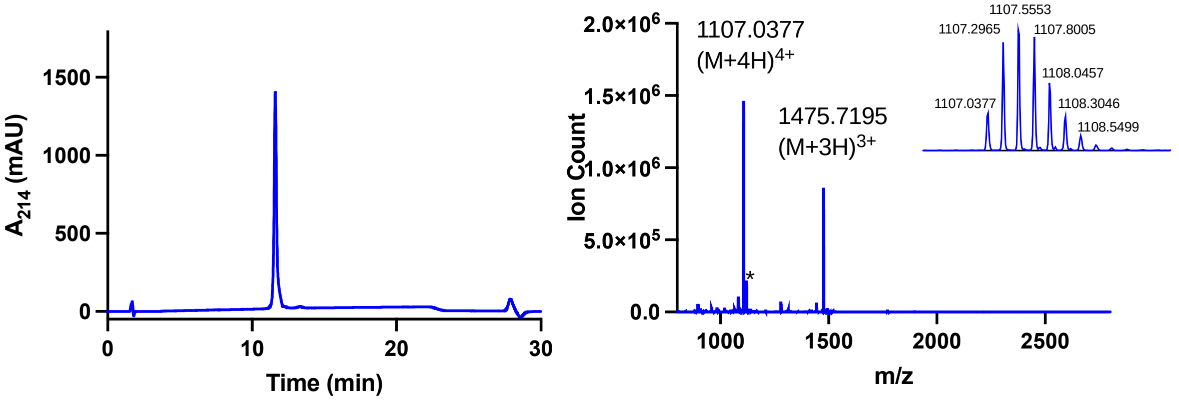

Figure S9. Characterisation of TAD1$_{1-39}$ pS9 acyl hydrazide peptide (precursor to **1b**) by RP-HPLC (left) and ESI-HRMS (right). HPLC purity >90% t_R= 11.6 min with gradient A, predicted: 4424.1465 Da, observed: 4424.1217 Da, peak denoted with an asterisk (*) is ammonium chloride adduct.

TAD1$_{1\text{-}39}$ NHNH$_2$ pS15

Synthesised on a 0.099 mmol scale, 4.9% isolated yield, 21.6 mg, special amino acids: Fmoc-Pra-OH (position 1), Fmoc-Pro-Pro-OH (position 11-12); see Table S1 for adjustments of the synthetic procedure.

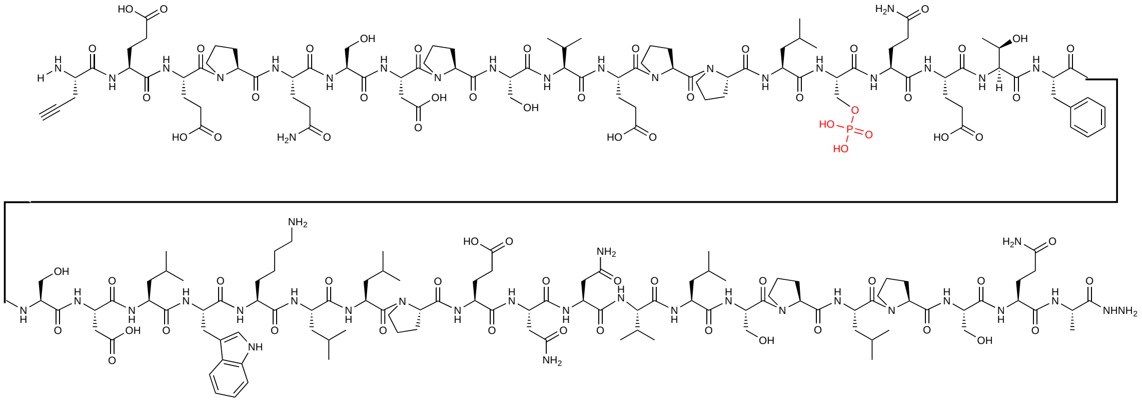

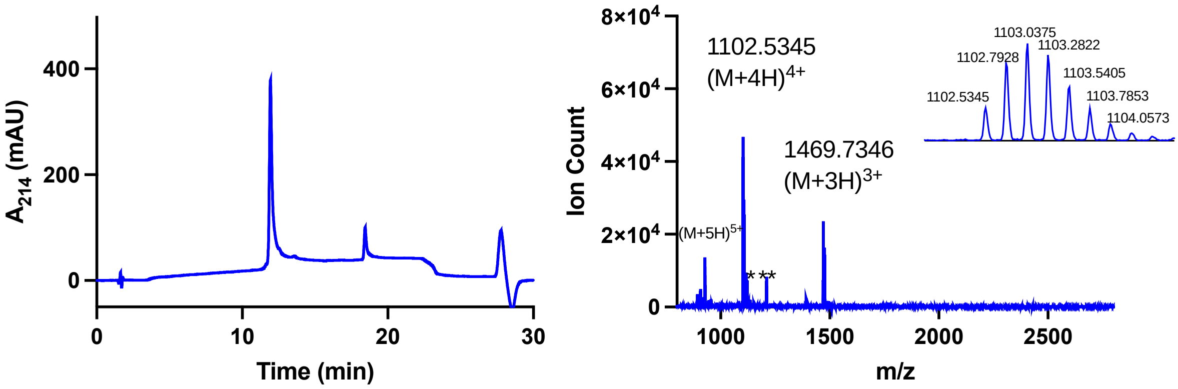

Figure S10. Characterisation of TAD1$_{1\text{-}39}$ Pra1, pS15 acyl hydrazide peptide (precursor to **1c**) by RP-HPLC (left) and ESI-HRMS (right). HPLC purity >80% t_R= 11.9 min with gradient A, predicted: 4406.0995 Da, observed: 4406.1089 Da, peak denoted with an asterisk (*) is ammonium chloride adduct, peak denoted with double asterisk (**) is TAD1$_{10\text{-}39}$ caused by cleavage of N-terminal sequence 7/8 (D-P sequence junction).

TAD1$_{1-39}$ NHNH$_2$ pT18

Synthesised on a 0.100 mmol scale, 20.0% isolated yield, 88.5 mg. See Table S1 for adjustments of the synthetic procedure.

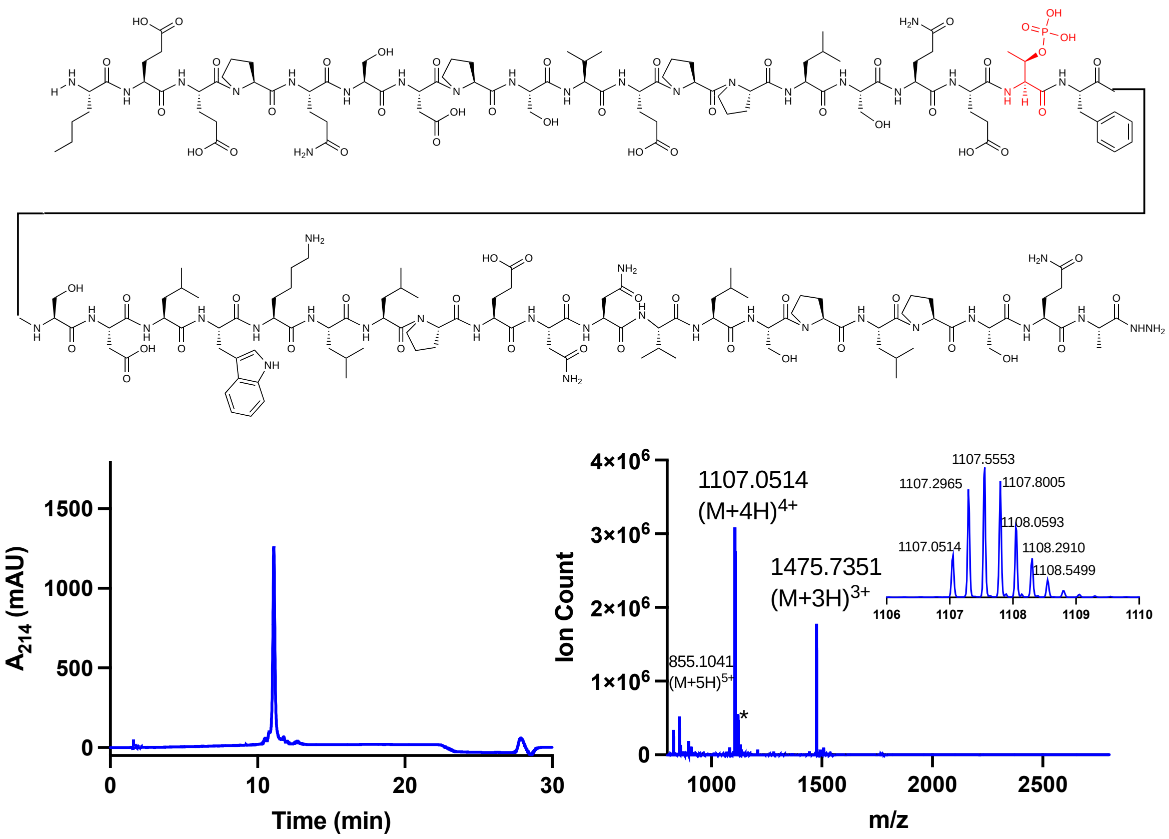

Figure S11. Characterisation of TAD1$_{1-39}$ pT18 acyl hydrazide peptide (precursor to **1d**) by RP-HPLC (left) and ESI-HRMS (right). HPLC purity >90% t_R= 11.1 min with gradient A, predicted: 4424.1465 Da, observed: 4424.1765 Da, peak denoted with an asterisk (*) is ammonium chloride adduct.

TAD1$_{1-39}$ NHNH$_2$ pS20

Synthesised on a 0.0675 mmol scale, 3.3% isolated yield, 8.7 mg. See Table S1 for adjustments of the synthetic procedure.

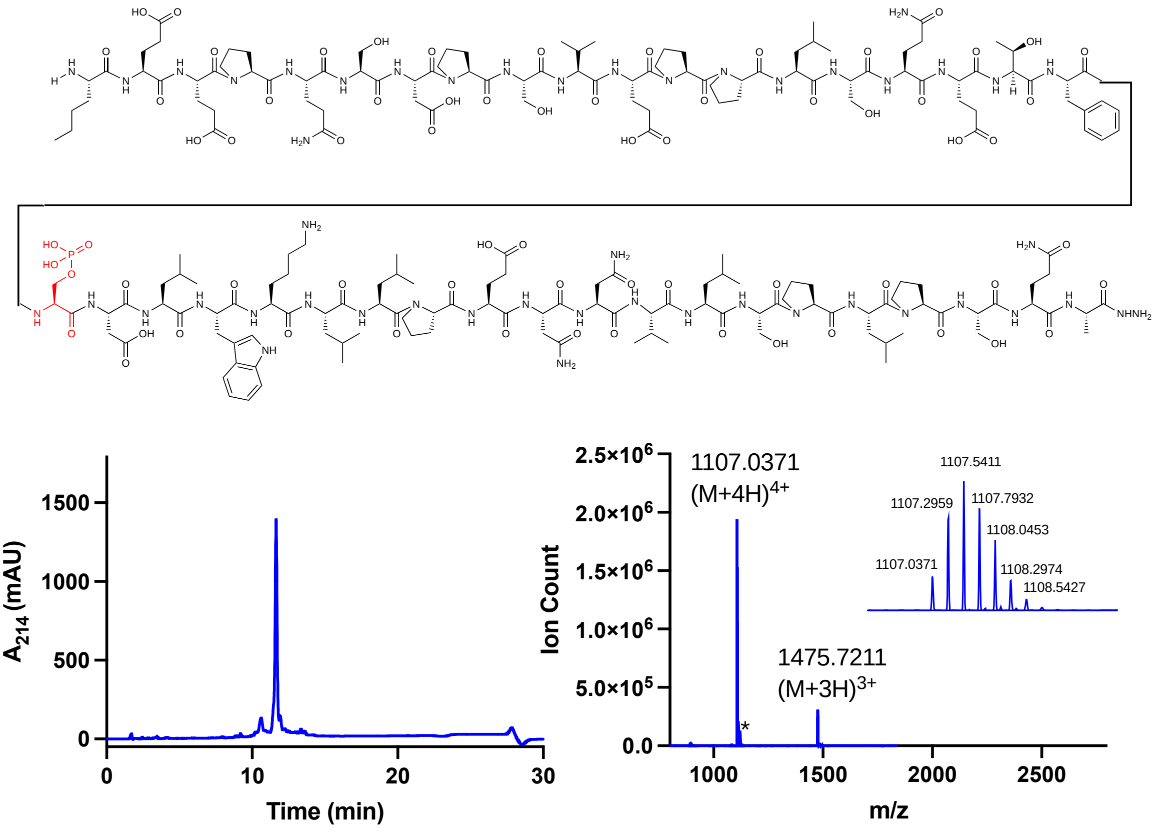

Figure S12. Characterisation of TAD1$_{1-39}$ pS20 acyl hydrazide peptide (precursor to **1e**) by RP-HPLC (left) and ESI-HRMS (right). HPLC purity >75% t_R= 11.7 min with gradient A, predicted: 4424.1465 Da, observed: 4424.1193 Da, peak denoted with an asterisk (*) is ammonium chloride adduct.

TAD1$_{1\text{-}39}$ NHNH$_2$ pS33

Synthesised on scale 0.100 mmol, 5.0% isolated yield, 22.1 mg, special amino acids: Fmoc-Pro-Pro-OH. See Table S1 for adjustments of the synthetic procedure.

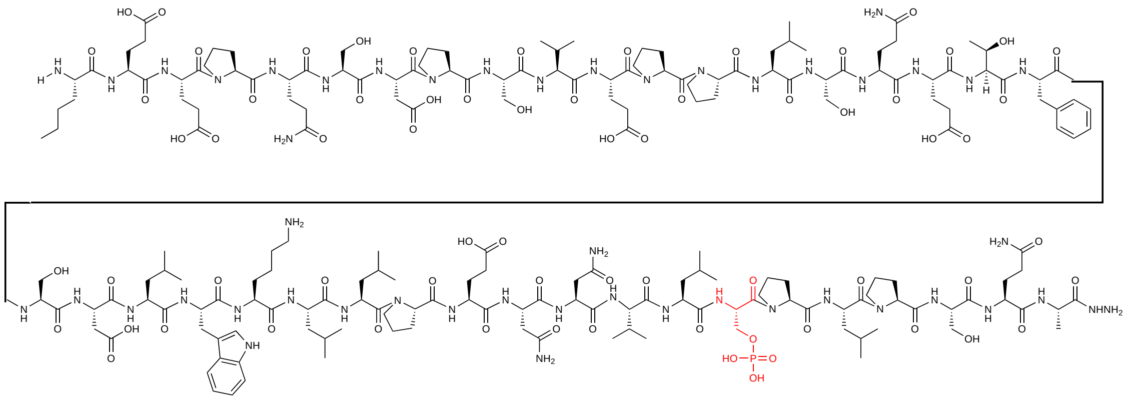

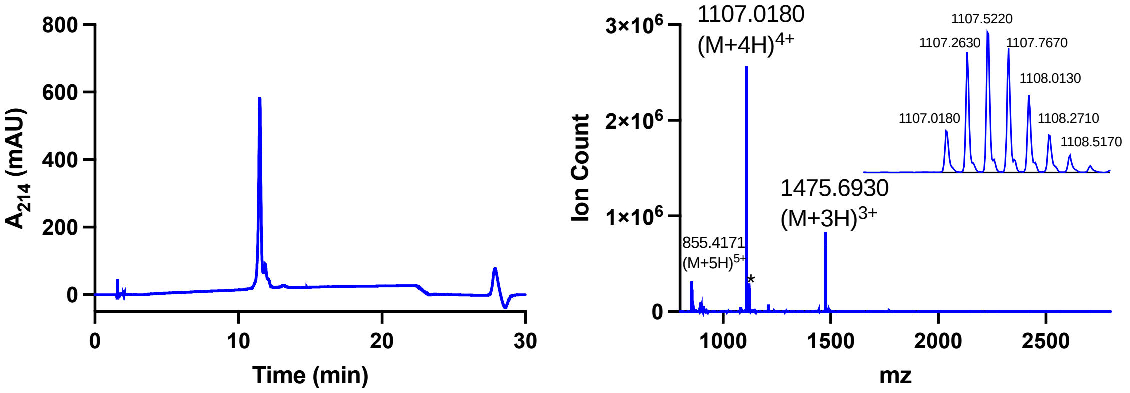

Figure S13. Characterisation of TAD1$_{1\text{-}39}$ pS33 acyl hydrazide peptide (precursor to **1f**) by RP-HPLC (left) and ESI-HRMS (right). HPLC purity >80% t_R= 11.5 min with gradient A, predicted: 4424.1465 Da, observed: 4424.0429 Da, peak denoted with an asterisk (*) is ammonium chloride adduct.

TAD1$_{1\text{-}39}$ NHNH$_2$ pS37

Synthesised on a 0.135 mmol scale, 1.0% isolated yield, 6.0 mg, see Table S1 for adjustments of the synthetic procedure.

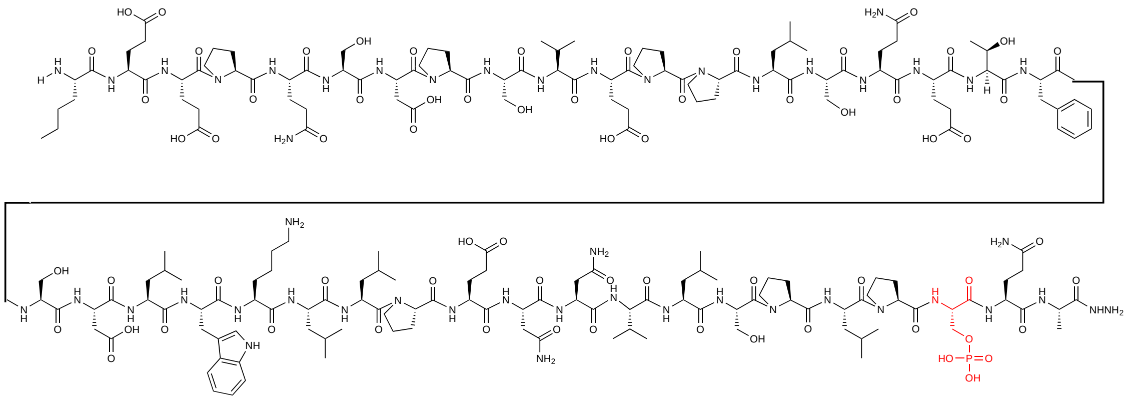

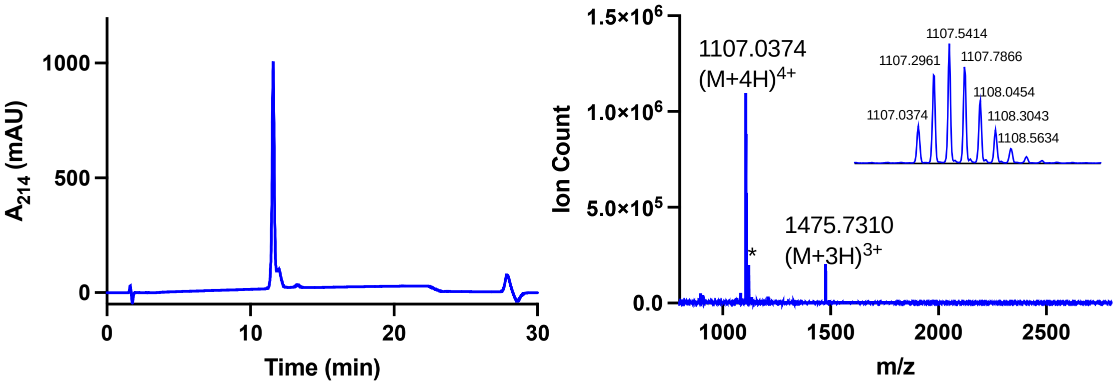

Figure S14. Characterisation of TAD1$_{1\text{-}39}$ pS37 acyl hydrazide peptide (precursor to **1g**) by RP-HPLC (left) and ESI-HRMS (right). HPLC purity >80% t_R= 11.6 min with gradient A, predicted: 4424.1465 Da, observed: 4424.1205 Da, peak denoted with an asterisk (*) is ammonium chloride adduct.

TAD1$_{1\text{-}39}$ NHNH$_2$ pS15pT18pS20

Synthesised on a 0.133 mmol scale, 1.3% isolated yield, 8.2 mg, see Table S1 for adjustments of the synthetic procedure.

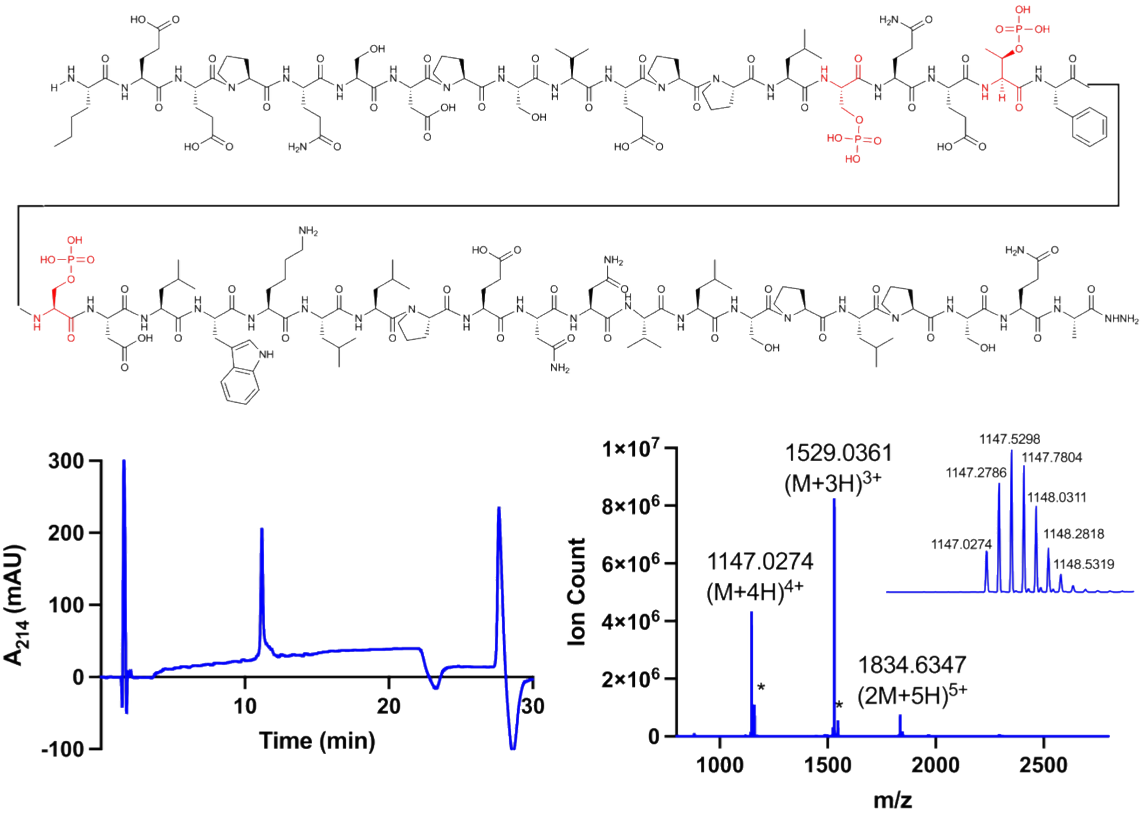

Figure S15. Characterisation of TAD1$_{1\text{-}39}$ pS15pT18pS20 acyl hydrazide peptide (precursor to **1h**) by RP-HPLC (left) and ESI-HRMS (right). HPLC purity >80% t_R= 11.2 min with gradient A, predicted: 4584.0792 Da, observed: 4584.0805 Da, peak denoted with an asterisk (*) is ammonium chloride adduct.

Expression and purification of p53ΔN

Protein Expression

p53ΔN was expressed as His_6-SUMO tag fusion from a pET3a plasmid in *E. coli* BL21 (DE3) via autoinduction following the methods described previously.[1,5] The plasmid was transformed into calcium competent *E. coli* and transformants were selected on 100 µg/ml ampicillin LB agar. A single colony was picked and grown overnight at 37 °C in MDG medium (50 mM NH_4Cl, 25 mM Na_2HPO_4, 25 mM KH_2PO_4, 5 mM Na_2SO_4, 2 mM $MgSO_4$, 0.5% glucose, 0.25% aspartate, 100 µg/ml ampicillin). The overnight bacterial culture was diluted 1:1000 in auto-induction medium (1% tryptone, 0.5% yeast extract, 50 mM NH4Cl, 25 mM Na_2HPO_4, 25 mM KH_2PO_4, 5 mM Na_2SO_4, 2 mM $MgSO_4$, 0.5% glycerol, 0.8% glucose, 0.2% α-lactose monohydrate, 100 µg/ml ampicillin) and grown overnight at 37 °C, 220 RPM. The overnight growth was centrifuged at 4 °C for 30 min and bacterial pellets were saved at -80 °C for future processing.

Inclusion Body Isolation

His_6-SUMO-p53ΔN were isolated from inclusion bodies from the autoinduction expression according to previously published methods. Bacterial pellets were resuspended in lysis buffer (0.1 M Tris-HCl, 1 mM EDTA) and digested with lysozyme (1.5 mg per 1 g of cells) for 30 min on ice. The lysates were further disrupted by sonication with a microtip (40% amplitude, 8 min total treatment, 5 sec on, 10 sec off) on ice, followed by Cell Disruptor treatment (Constant Systems Ltd.) at 25 kpsi, 4 °C and repeated 3 times. The resulting lysate was incubated with DNase (10 µg/ml, 3 mM $MgCl_2$) for 30 min at RT with gentle shaking. DNase was quenched by the addition of 0.5 volume of quench buffer (60 mM EDTA, 6 % Triton X-100, 1.5 M NaCl pH 7). The solution was centrifuged at 30,000 x g at 4 °C for 60 min. The pellet containing inclusion bodies was washed twice with buffers of decreasing ionic strength and triton-x concentration. The washed pellet was resuspended in an argon degassed solubilization buffer (6 M Guanidinium chloride, 0.1 M Tris-HCl, 50 mM DTT, pH 8) and left rotating for 1 hr. After incubation with solubilization buffer, the solution was acidified to pH 2-3 with HCl and centrifuged at 30,000 x g for 60 min at 4 °C. The supernatant, containing the solubilized His_6-SUMO-p53ΔN was immediately desalted on PD-10 disposable columns with buffer exchanged to argon degassed 30% MeCN

(0.1% formic acid) and lyophilized for long-term storage. Aliquots of desalted protein were analyzed on RP-HPLC (20-60% B, RT, 30 min), as well as on ESI-HRMS. Aliquots from the expression and inclusion bodies solubilization were mixed with 4x SDS loading dye and saved at -20 °C for further analysis by SDS-PAGE.

Cleavage of His_6-SUMO tag and purification

Fractions collected after the desalting stage were resuspended to a concentration of 10-20 mg/ml in argon degassed solubilization buffer (6 M Guanidinium chloride, 0.1 M Tris-HCl, 50 mM DTT, pH 8) and left for 30 min standing at RT. Following the incubation, the solubilized material was diluted to 0.5-1 mg/mL with argon degassed reaction buffer (150 mM NaCl, 50 mM Tris-HCl, 1.4 M Urea, 100 mM arginine; final conc. guanidinium chloride 0.2 M, final conc. DTT 0.75 mM). ULP-1 (prepared in-house) was added to the reaction buffer to a final concentration of 0.02 mg/mL. The reaction was incubated at RT with shaking at 50 RPM for 1h. After 1h the reaction mixture was loaded with a peristaltic pump at 1 mL/min onto a pre-equilibrated reverse nickel column (HisTrap™ High Performance, 5 ml, Cytiva®). The flow through was collected and DTT was added to the final concentration of 50 mM and left incubating for 30 min at RT. After the reduction, p53ΔN was precipitated by the addition of 100% TCA (w/v) (4 V reaction: 1 V TCA) and incubated for 10 min on ice. The precipitate was centrifuged for 5 min at 14,000 RPM at 4 °C, resuspended in 6 M GdmCl, 0.1 M Tris and desalted using PD-10 column as described above. Desalted protein was lyophilized. Following freeze-drying, the crude was redissolved in 10 mL 25% MeCN per 1 L of expression culture, filtered and purified on preparative HPLC (C3 column) using gradient E.

Gradient E: 25-45% B over 20 min at 20 mL/min, RT

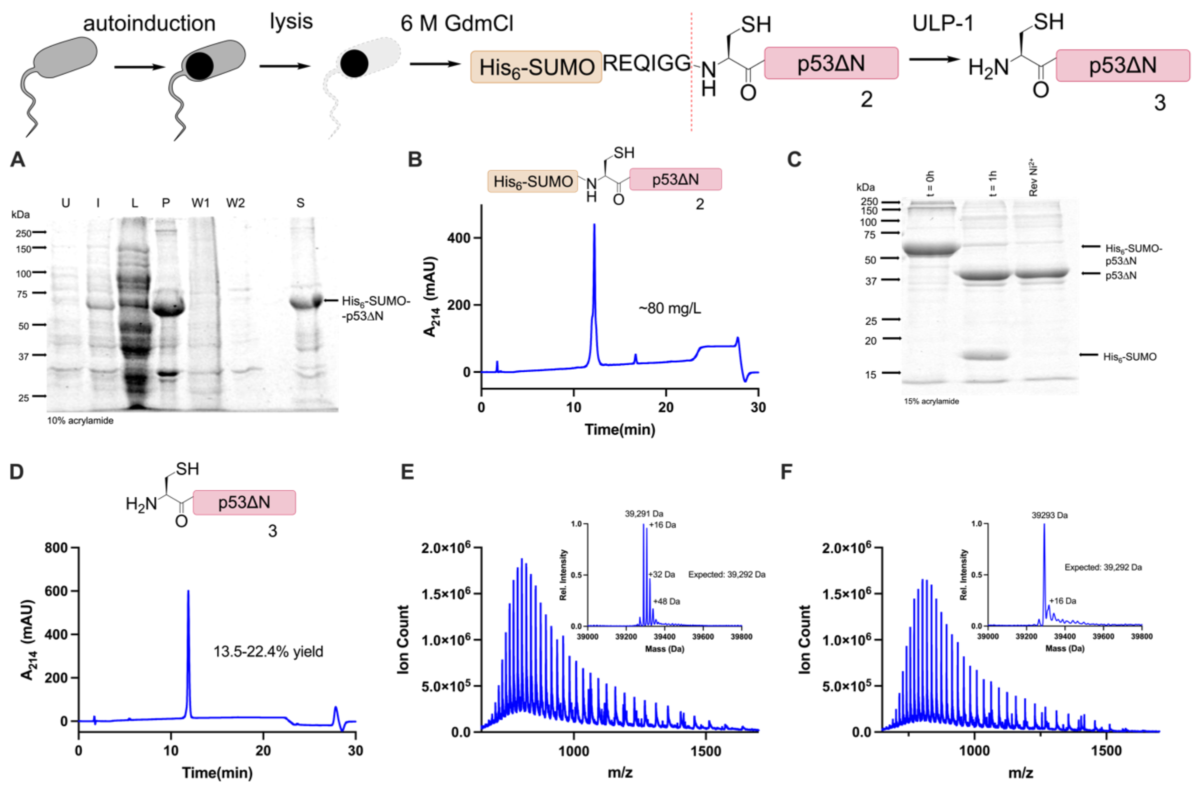

Figure S16. Recombinant production of p53ΔN M40C. His6-SUMO-p53ΔN M40C was expressed in BL21 cells under ampicillin antibiotic selection and purified from inclusion bodies to generate His6-SUMO-p53ΔN. **(A)** Expression gel with inclusion body extraction (10% acrylamide), the band corresponding to the protein can be seen between the 50 and 75 kDa marks; lanes: U- uninduced cells, I- induced cells, L- cell lysate soluble fraction, P- cell lysate pellet, W1- wash1 of the pellet, W2- wash2 of the pellet, S- pellet solubilised in 6M GdmCl; (**B**) RP-HPLC of guanidinium chloride solubilized His6-SUMO-p53ΔN, estimating 80 mg/L of expression culture, (**C)**15% acrylamide gel of Ulp-1 cleavage of the His6-SUMO tag, with the reaction running to completion within an hour at RT, and successful removal of the His6-SUMO tag with the reverse nickel purification step, (**D**) RP-HPLC of purified p53ΔN M40C with associated yields, (**E)**ESI- HRMS of oxidized (+16 Da), purified p53ΔN M40C, the inset shows the deconvoluted spectrum of the protein peak with multiple oxidations, (**F)** ESI- HRMS of purified p53ΔN M40C, the inset shows the deconvoluted spectrum of the protein peak; only preparations of p53ΔN presented in F were used for downstream NCL reactions.

Synthesis of full-length phospho-p53 variants via Native Chemical Ligation

Methods for Native Chemical Ligation

Procedure 1

TAD1 peptide hydrazide (1 μmol, 4 mg) was dissolved in 200 μL of argon degassed Ligation buffer pH 3 (50 mM NaH_2PO_4, 6 M Gdm·HCl,1.5mM EDTA) and incubated in ice-salt bath at -15 °C for 5 min. Then, 4 μL of 5 M $NaNO_2$ were added and reaction was agitated at -15 °C for 15 min. Following the *in situ* oxidation of hydrazide to azide, 42 μL MPAA of 2 M MPAA were added simultaneously with NaOH to increase pH to approximately 7. Immediately after, p53ΔN (0.5 μmol, 20 mg) dissolved in 200 μL of argon degassed Ligation buffer pH 3 was added. The reaction was warmed up to room temperature and let to agitate for 1h. To monitor the reaction, 4 μL were removed and incubated with 1 μL of 1 M DTT for 5 min, followed by further dilution with 35 μL of argon degassed Ligation buffer pH 3. Then, 10 μL were injected onto C3 RP-HPLC column and analyzed using gradient A. Peaks were collected for ESI-HRMS analysis. Once the reaction did not proceed further it was quenched by addition of 50 mM DTT, incubated for 30 min and subsequently acidified and injected onto semi-preparative C3 column and purified using gradient D. Fractions (2 mL) were collected when A_{214} > 200 mAu, and subsequently lyophilized. Fractions were analyzed on RP-HPLC using gradient A and on ESI-HRMS with protein-specific settings. Fractions containing pure product were pooled and lyophilized.

Procedure 2

TAD1 peptide hydrazide (1 μmol, 4 mg) was dissolved in 200 μL of argon degassed Ligation buffer pH 3 (50 mM NaH_2PO_4, 6 M Gdm·HCl,1.5mM EDTA) and incubated in ice-salt bath at -15 °C for 5 min. Then, 4 μL of 5 M $NaNO_2$ were added, and reaction was agitated at -15 °C for 15 min. Following the *in-situ* oxidation of hydrazide to azide, 42 μL MPAA of 2 M MPAA were added simultaneously with NaOH to increase pH to approximately 7. Immediately after, p53ΔN (0.5 μmol, 20 mg) dissolved in 200 μL of argon degassed Ligation buffer pH 3 was added. The reaction was warmed up to room temperature and let to agitate for 1h. To monitor the reaction, 4 μL were removed and

incubated with 1 μL of 1 M DTT for 5 min, followed by further dilution with 35 μL of argon degassed Ligation buffer pH 3. Then, 10 μL were injected onto C3 RP-HPLC column and analyzed using gradient A. Peaks were collected for ESI-HRMS analysis. Once the reaction did not proceed further it was quenched by addition of **200 mM DTT**, incubated for 30 min and subsequently acidified and injected onto semi-preparative C3 column and purified using gradient D. Fractions (2 mL) were collected when A_{214} > 200 mAu, and subsequently lyophilized. Fractions were analysed on RP-HPLC using gradient A and on ESI-HRMS with protein-specific settings. Fractions containing pure product were pooled and lyophilized.

Procedure 3

TAD1 peptide hydrazide (1 μmol, 4 mg) was dissolved in 200 μL of argon degassed Ligation buffer pH 3 (50 mM NaH_2PO_4, 6 M Gdm·HCl,1.5mM EDTA) and incubated in ice-salt bath at -15 °C for 5 min. Then, 4 μL of 5 M $NaNO_2$ were added, and reaction was agitated at -15 °C for 15 min. Following the *in-situ* oxidation of hydrazide to azide, 42 μL MPAA of 2 M MPAA were added simultaneously with NaOH to increase pH to approximately 7. Immediately after, p53ΔN (0.5 μmol, 20 mg) dissolved in 200 μL of argon degassed Ligation buffer pH 3 was added. The reaction was warmed up to room temperature and let to agitate for 1h. **The reaction was conducted with an argon filled ballon on top of the HPLC vial housing the reaction.** To monitor the reaction, 4 μL were removed and incubated with 1 μL of 1 M DTT for 5 min, followed by further dilution with 35 μL of argon degassed Ligation buffer pH 3. Then, 10 μL were injected onto C3 RP-HPLC column and analyzed using gradient A. Peaks were collected for ESI-HRMS analysis. Once the reaction did not proceed further it was quenched by addition of 200 mM DTT, incubated for 30 min and subsequently acidified and injected onto semi-preparative C3 column and purified using gradient D. Fractions (2 mL) were collected when A_{214} > 200 mAu, and subsequently lyophilized. Fractions were analyzed on RP-HPLC using gradient A and on ESI-HRMS with protein-specific settings. Fractions containing pure product were pooled and lyophilized.

Procedure 4

TAD1 peptide hydrazide (1 μmol, 4 mg) was dissolved in 200 μL of argon degassed Ligation buffer pH 3 (50 mM NaH_2PO_4, 6 M Gdm·HCl,1.5mM EDTA) and incubated in ice-salt bath at -15 °C for 5 min. Then, 4 μL of 5 M $NaNO_2$ were added and reaction was agitated at -15 °C for 15 min. Following the *in-situ* oxidation of hydrazide to azide, 42 μL MPAA of 2 M MPAA were added simultaneously with NaOH to increase pH to approximately 7. Immediately after, p53ΔN **(0.25 μmol, 10 mg)** dissolved in 200 μL of argon degassed Ligation buffer pH 3 was added. The reaction was warmed up to room temperature and let to agitate for 1h. **TCEP (50 mM final conc.) was added to reaction as a reducing agent.** The reaction was conducted with an argon filled ballon on top of the HPLC vial housing the reaction. To monitor the reaction, 4 μL were removed and incubated with 1 μL of 1 M DTT for 5 min, followed by further dilution with 35 μL of argon degassed Ligation buffer pH 3. Then, 10 μL were injected onto C3 RP-HPLC column and analyzed using gradient A. Peaks were collected for ESI-HRMS analysis. Once the reaction did not proceed further it was quenched by addition of **200 mM cysteamine and further 50 mM TCEP**, incubated for 30 min and subsequently acidified and injected onto semi-preparative C3 column and purified using gradient D. Fractions (2 mL) were collected when A_{214} > 200 mAu, and subsequently lyophilized. Fractions were analyzed on RP-HPLC using gradient A and on ESI-HRMS with protein-specific settings. Fractions containing pure product were pooled and lyophilized.

Procedure 5

TAD1 peptide hydrazide (1 μmol, 4 mg) was dissolved in 200 μL of argon degassed Ligation buffer pH 3 (**200 mM NaH_2PO_4**, 6 M Gdm·HCl,1.5mM EDTA) and incubated in ice-salt bath at -15 °C for 5 min. Then, 4 μL of 5 M $NaNO_2$ were added, and reaction was agitated at -15 °C for 15 min. Following the *in-situ* oxidation of hydrazide to azide, 42 μL MPAA of 2 M MPAA were added simultaneously with NaOH to increase pH to approximately 7. Immediately after, p53ΔN (0.25 μmol, 10 mg) dissolved in 200 μL of argon degassed Ligation buffer pH 3 was added. The reaction was warmed up to room temperature and let to agitate for 1h. TCEP (50 mM final conc.) was added to reaction as a reducing agent. The reaction was conducted with an argon filled ballon on top of

the HPLC vial housing the reaction. To monitor the reaction, 4 μL were removed and incubated with 1 μL of 1 M DTT for 5 min, followed by further dilution with 35 μL of argon degassed Ligation buffer pH 3. Then, 10 μL were injected onto C3 RP-HPLC column and analyzed using gradient A. Peaks were collected for ESI-HRMS analysis. Once the reaction did not proceed further it was quenched by addition of 200 mM cysteamine and further 50 mM TCEP, incubated for 30 min and subsequently acidified and injected onto semi-preparative C3 column and purified using gradient D. Fractions (2 mL) were collected when A_{214} > 200 mAu, and subsequently lyophilized. Fractions were analyzed on RP-HPLC using gradient A and on ESI-HRMS with protein-specific settings. Fractions containing pure product were pooled and lyophilized.

Characterization data for full-length p53 variants

<u>$p53_{1-393}$ pS6 (**4a**)</u>

Reaction scale in reference to p53ΔN: 0.5 μmol, TAD1 peptide hydrazide used in the reaction: 1 μmol or 2eq., isolated yield in reference to p53ΔN: 19%, 0.096 μmol (4.2 mg), t_R = 13.0 min using gradient A, expected: 43687.0 Da, observed: 43,686.0 Da; Prepared using procedure 2.

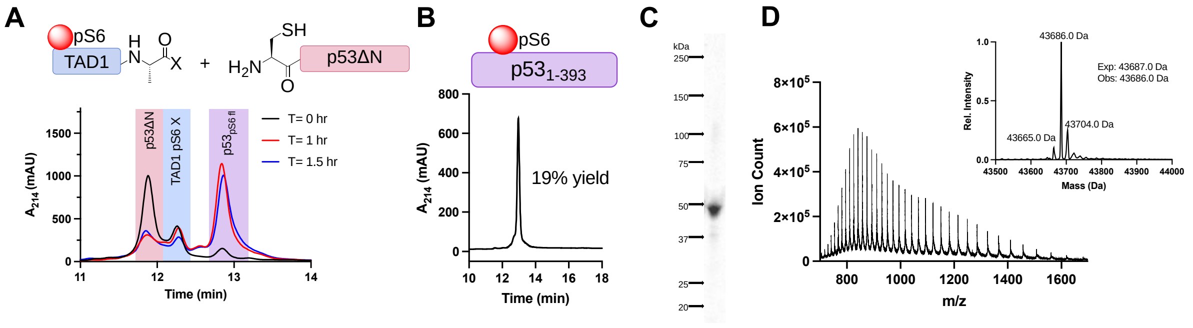

Figure S17. Preparation and characterisation of $p53_{1-393}$ pS6 via NCL. (A) Reaction monitoring by RP-HPLC, blue shaded area is TAD1 pS6 where X = DTT in the RP-HPLC and MPAA in the reaction, pink shaded area is p53ΔN, purple shaded area is $p53_{1-393}$ pS6 (**4a**). (B, C) Analysis of the isolated product by RP-HPLC (B) and SDS-PAGE (C; 1 μg loading). (D) ESI-HRMS of the isolated product with inset showing deconvoluted spectrum.

$p53_{1\text{-}393}$ pS9 (**4b**)

Reaction scale in reference to p53ΔN: 0.5 µmol, TAD1 peptide hydrazide used in the reaction: 1 µmol or 2eq., isolated yield in reference to p53ΔN: 30%, 0.151 µmol (6.6 mg), t_R = 13.0 min using gradient A, expected: 43687.0 Da, observed: 43,686.0 Da. Prepared with Procedure 3.

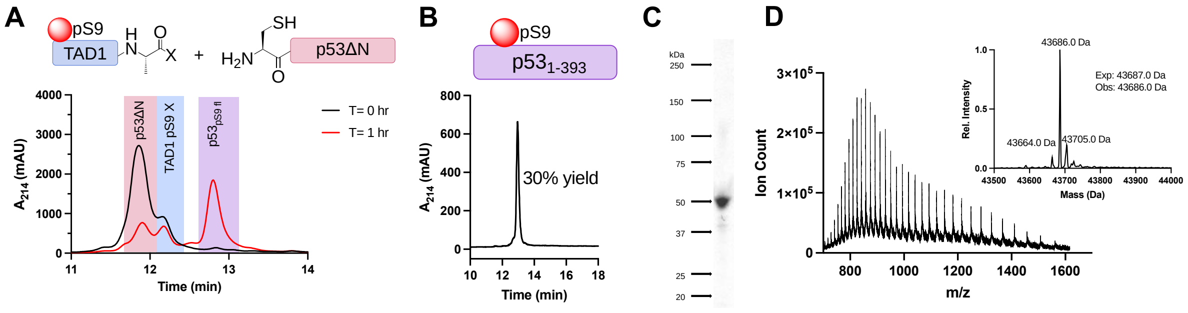

Figure S18. Preparation and characterisation of $p53_{1\text{-}393}$ pS9 via NCL. Prepared using procedure 3. (A) Reaction monitoring by RP-HPLC, X = DTT and X = OH, pink shaded area is p53ΔN, purple shaded area is $p53_{1\text{-}393}$ pS9 (**4b**). (B, C) Analysis of the isolated product by RP-HPLC (B) and SDS-PAGE (C; 1 µg loading). (D) ESI-HRMS of the isolated product with inset showing deconvoluted spectrum.

p53$_{1-393}$ Pra1,pS15 (**4c**)

Reaction scale in reference to p53ΔN: 0.138 µmol, TAD1 peptide hydrazide used in the reaction: 0.5 µmol or 3.6 eq., isolated yield in reference to p53ΔN: 25%, 0.034 µmol (1.5 mg), t_R = 12.8 min using gradient A, expected: 43669.0 Da, observed: 43,668.0 Da; Prepared using procedure 5.

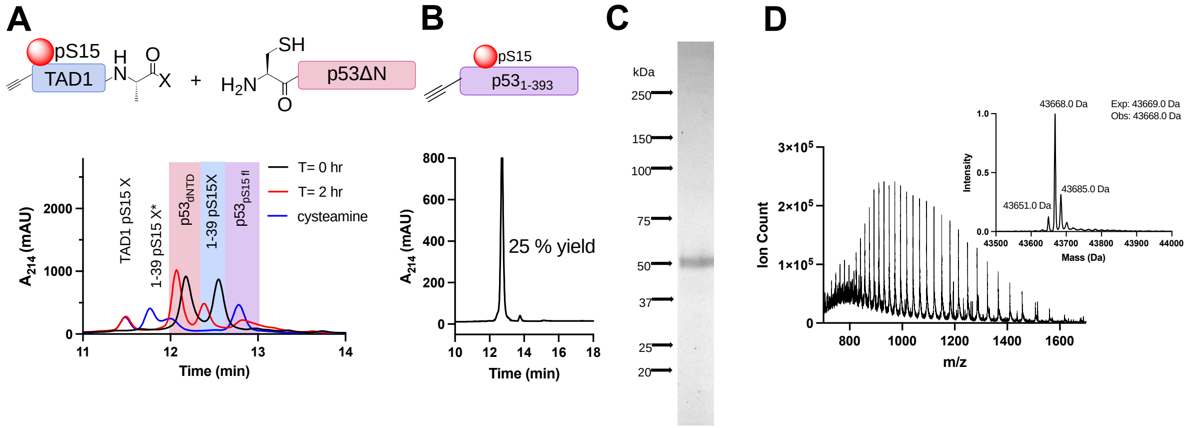

Figure S19. Preparation and characterization of p53$_{1-393}$ pS15 via NCL. (A)- reaction monitoring by RP-HPLC, grey shaded area is TAD1 pS15: X = OH; TAD1 pS15 X*: X = cysteamine; blue shaded area is TAD1 pS15 (**1c**) X= MPAA, pink shaded area is p53ΔN, purple shaded area is p53$_{1-393}$ pS15 (**4c**). (B, C) Analysis of the isolated product by RP-HPLC (B) and SDS-PAGE (C; 1 µg loading). (D) ESI-HRMS of the isolated product with inset showing deconvoluted spectrum.

p53$_{1-393}$ pT18 (**4d**)

Reaction scale in reference to p53ΔN: 0.184 µmol, TAD1 used in the reaction: 1 µmol or 5.4 eq., isolated yield in reference to p53ΔN: 57.1%, 0.107 µmol (4.7 mg), t_R = 12.8 min using gradient A, expected: 43687.0 Da, observed: 43,686.0 Da; Prepared using procedure 4.

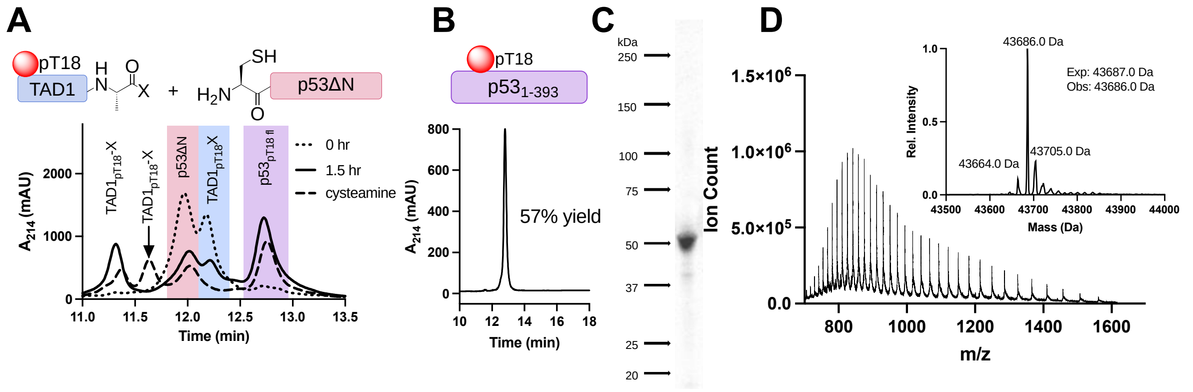

Figure S20. Preparation and characterisation of p53$_{1-393}$ pT18 via NCL. (A) reaction monitoring by RP-HPLC: grey shaded area is TAD1 pT18 (**1d**): X = OH; blue shaded area is TAD1 pT18: X = MPAA; indicated with an arrow is TAD1 pT18: X = cysteamine, pink shaded area is p53ΔN; purple shaded area is p53 pT18 (**4d**). (B, C) Analysis of the isolated product by RP-HPLC (B) and SDS-PAGE (C; 1 µg loading). (D) ESI-HRMS of the isolated product with inset showing deconvoluted spectrum.

p53$_{1-393}$ pS20 (**4e**)

Reaction scale in reference to p53ΔN: 0.5 µmol, TAD1 peptide hydrazide used in the reaction: 1 µmol or 2eq., isolated yield in reference to p53ΔN 14% 0.071 µmol (3.1 mg), t_R = 13.0 min using gradient A, expected: 43687.0 Da, observed: 43,685.0 Da. Prepared using procedure 1.

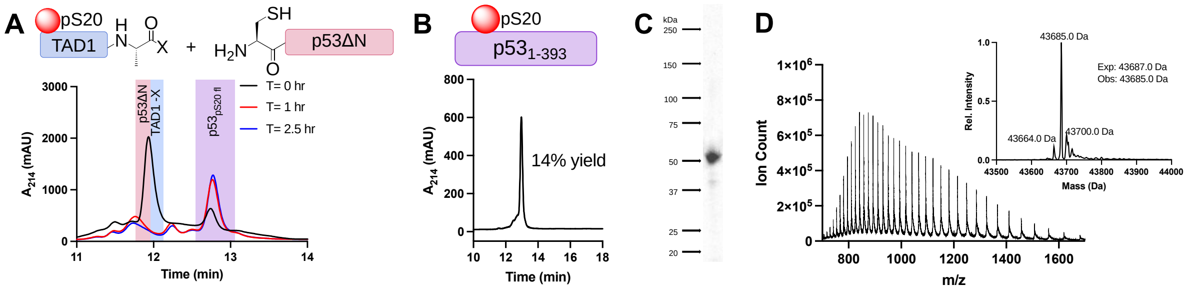

Figure S21. Preparation and characterisation of p53$_{1-393}$ pS20. (A) Reaction monitoring by RP-HPLC, blue shaded area is TAD1-X where X = DTT; pink shaded area is p53ΔN; purple shaded area is p53$_{1-393}$ pS20. (B, C) Analysis of the isolated product by RP-HPLC (B) and SDS-PAGE (C; 1 µg loading). (D) ESI-HRMS of the isolated product with inset showing deconvoluted spectrum.

p53$_{1-393}$ pS33 (**4f**)

Reaction scale in reference to p53ΔN: 0.245 μmol, TAD1 used in the reaction: 2 μmol or 8.1 eq., isolated yield in reference to p53ΔN: 37%, 0.091 μmol (4.0 mg), t_R = 12.8 min using gradient A, expected: 43687.0 Da, observed: 43,686.0 Da; Prepared using procedure 4.

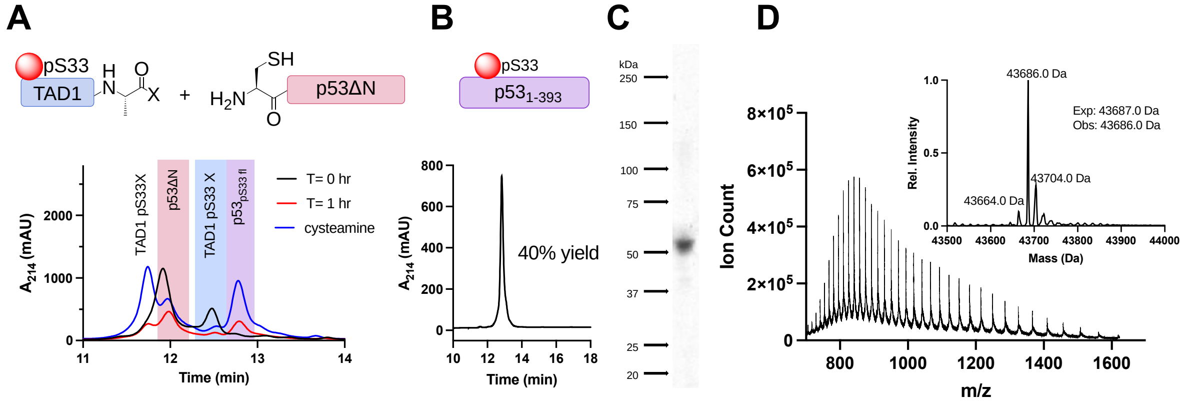

Figure S22. Preparation and characterisation of p53$_{1-393}$ pS33 via NCL. (A) Reaction monitoring by RP-HPLC, blue shaded area is TAD1-X (**1f**) where X = MPAA; grey shaded area is TAD1 pS33 X where X = OH; pink shaded area is p53ΔN; purple shaded area is p53$_{1-393}$ pS33 (**4f**). (B, C) Analysis of the isolated product by RP-HPLC (B) and SDS-PAGE (C; 1 μg loading). (D) ESI-HRMS of the isolated product with inset showing deconvoluted spectrum.

p53$_{1\text{-}393}$ pS37 (**4g**)

Reaction scale in reference to p53ΔN: 0.190 µmol, TAD1 used in the reaction: 0.7 µmol or 3.7 eq., isolated yield in reference to p53ΔN: 21.0%, 0.040 µmol (1.8 mg), t_R = 12.9 min using gradient A, expected: 43687.0 Da, observed: 43,686.0 Da. Prepared using procedure 5.

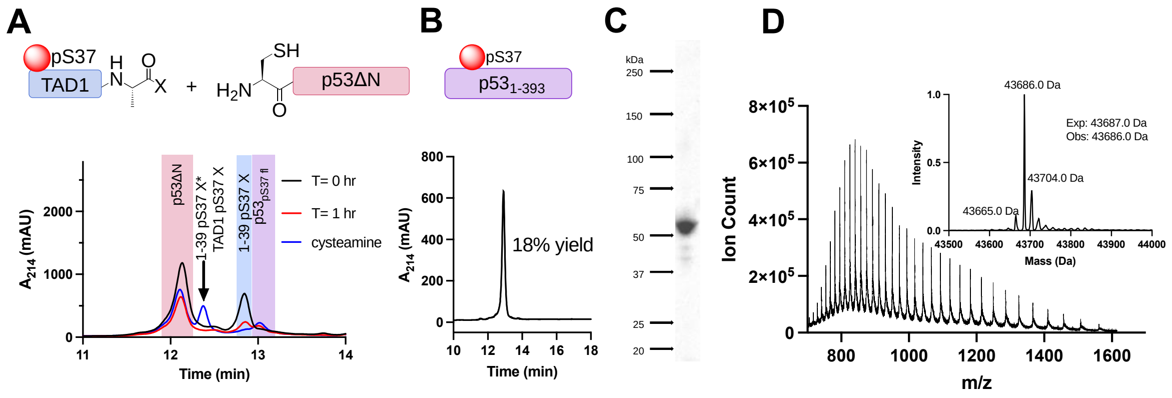

Figure S23. Preparation and characterisation of p53$_{1\text{-}393}$ pS37. (A) Reaction monitoring by RP-HPLC where blue shaded area is TAD1-X (**1g**) where X = MPAA; grey shaded area is TAD1 pS37 X where X = OH; an arrow indicates TAD1 pS37 X* where X = cysteamine; pink shaded area is p53ΔN (**3**); purple shaded area is p53$_{1\text{-}393}$ pS37 (**4g**). (B, C) Analysis of the isolated product by RP-HPLC (B) and SDS-PAGE (C; 1 µg loading). (D) ESI-HRMS of the isolated product with inset showing deconvoluted spectrum.

p53₁₋₃₉₃ pS15pT18pS20 (**4h**)

Reaction scale in reference to p53ΔN: 0.38 µmol, TAD1 used in the reaction: 0.8 µmol or 2.1 eq., isolated yield in reference to p53ΔN: 12.0%, 0.045 µmol (2.0 mg), t_R = 12.9 min using gradient A, expected: 43846.0 Da, observed: 43,846.5 Da. Prepared using procedure 2.

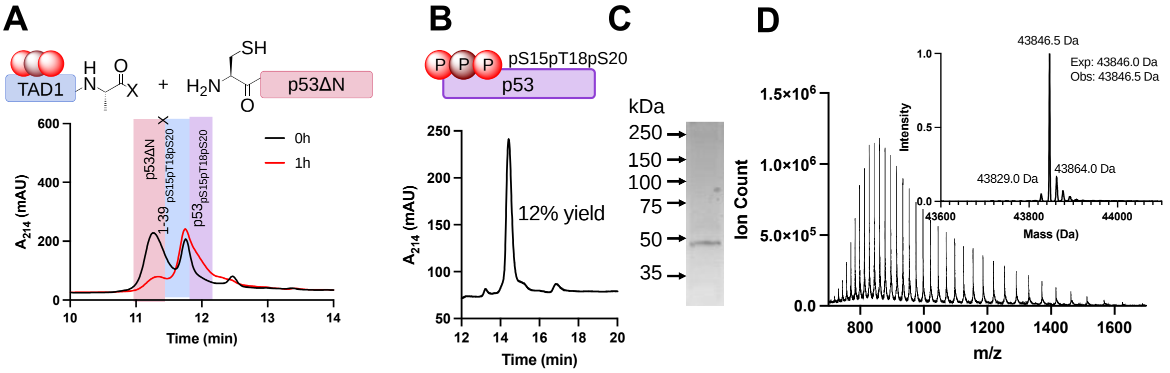

Figure S24. Preparation and characterisation of p53$_{1-393}$ pS15pT18pS20. (A) Reaction monitoring by RP-HPLC where blue shaded area is TAD1-X (**1h**) where X = MPAA; pink shaded area is p53ΔN (**3**); purple shaded area is p53$_{1-393}$ pS15pT18pS20 (**4h**). (B, C) Analysis of the isolated product by RP-HPLC (B) and SDS-PAGE (C; 50 ng loading). (D) ESI-HRMS of the isolated product with inset showing deconvoluted spectrum.

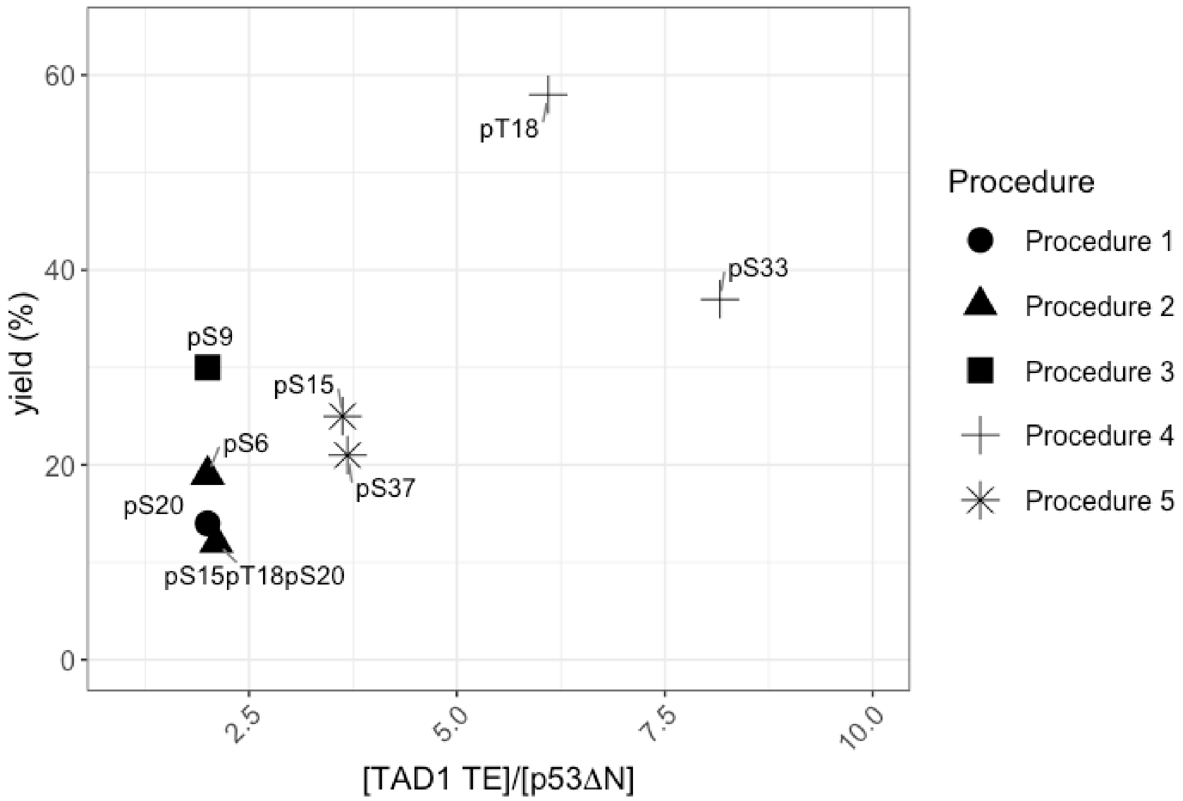

Procedure	DTT post reaction	Argon	50 mM TCEP in reaction	200 mM cysteamine treatment	200 mM buffer	p53ΔN (μmol)
**1**	50 mM	No	No	No	No	0.5
**2**	200 mM	No	No	No	No	0.5
**3**	200 mM	Yes	No	No	No	0.5
**4**	N/A	Yes	Yes	Yes	No	0.2
**5**	N/A	Yes	Yes	Yes	Yes	0.2

Figure S25. Summary of the effect of interventions on the NCL yield for p53.

Cross Validation of ‘designer’ phospho-p53 library and p53-specific antibodies by Western Blot

Materials and Methods

Samples (approx. 50-100 ng of modified p53) were loaded on to a polyacrylamide gel for SDS-PAGE. When made in-house, gels consisted of a 10 % resolving gel (10 % acrylamide, 375 mM Tris pH 8.8, 0.1 % SDS, 0.5 % TCE, 0.1 % APS and 0.1 % TEMED. Alternatively, 4-15 % stain-free precast gels (Bio-Rad) were also used. Gels were run at 180 V for 50-60 minutes in SDS running buffer (24.76 mM Tris base, 0.19 M glycine, 3.48 mM SDS). After stain-free imaging with 5-minutes activation, gels were soaked in transfer buffer (24.76 mM Tris base, 0.19 M glycine, 0.06 % SDS) for 15 minutes. Extra thick filter paper (Bio-Rad) and PVDF membranes (activated in methanol for 60 seconds) were also soaked transfer buffer for 15 minutes. Transfer was carried out using the for up to 40 min at 2.5A and 25V on the Trans-Blot Turbo Transfer System (Bio-Rad). When gels were imaged again post-transfer, this was achieved by stain-free imaging with no activation. Membranes were blocked in 3 % BSA in TBST, or 5% milk in TBST (19.8 mM Tris base, 0.15 M NaCl, 0.1 % Tween-20). See Table S2 for the summary of primary antibodies used in this study. Blocking was carried out at room-temperature for 1 hr. Membranes were incubated with primary antibodies, diluted in blocking solution according to manufacturer’s instructions, for approximately 16 hours at 4 ◦C. Membranes were washed with TBST for 5 minutes three times before 1 hr incubation at room-temperature with the relevant secondary antibody, diluted in TBST according to manufacturer’s instructions. Membranes were then washed with TBST for 5 minutes three times. Clarity ECL substrate (Biorad) was applied to the membrane before imaging using a Chemidoc Imaging System (Bio-Rad).

Table S2. Summary of antibodies used.

Antibody	Supplier	Catalogue	Host species	Blocking	Dilution	Exposure time
**α-phosSer6 p53 [Y179]**	Abcam	Ab32132	rabbit	3% BSA in TBS-T	1:1000	1s
**α-phosSer6 p53**	Abbexa	abx000209	rabbit	3% BSA in TBS-T	1:1000	2s
**α-phosSer9 p53**	CST	#9288	rabbit	3% BSA in TBS-T	1:1000	0.2s
**α-phosSer15 p53**	Abcam	Ab223868	rabbit	3% BSA in TBS-T	1:5000	60s
**α-phosThr18 p53**	ThermoFisher	PA5-12660	rabbit	3% BSA in TBS-T	1:1000	2s
**α-phosSer20 p53**	Abcam	Ab157454	rabbit	3% BSA in TBS-T	1:1000	2s
**α-phosSer33 p53**	Abcam	Ab75867	rabbit	3% BSA in TBS-T	1:3000	2s
**α-phosSer37 p53**	Abcam	Ab182164	rabbit	3% BSA in TBS-T	1:3000	0.2s
**α- p53 DO-1**	Abcam	Ab1101	mouse	5% milk in TBS-T	1:3000	60s
**α- p53 DO-1**	Santa Cruz	SC-126	mouse	3% BSA in TBS-T	1:1000	3s
**α- p53 Y5**	Abcam	Ab32049	rabbit	3% BSA in TBS-T	1:1000	3s
**α- p53 SP5**	Abcam	Ab16665	rabbit	3% BSA in TBS-T	1:1000	3s
**α- p53 Ab240**	Abcam	Ab26	mouse	3% BSA in TBS-T	1:1000	3s
**Goat Anti-Mouse IgG (H + L)-HRP conjugate**	BioRad	#1706516	Goat anti-mouse	See 1°	1:10,000	See 1°
**Goat Anti-Rabbit IgG (H + L)-HRP conjugate**	BioRad	#1706515	Goat Anti-rabbit	See 1°	1:10,000	See 1°

Quantitative Analysis of phos-p53 in Western Blot

Samples of p53 phospho-protein isoforms were loaded on self-cast 10% polyacrylamide SDS gels with loadings 25, 50 and 100 ng. Transfer and antibody incubation was performed as described above. Densitometric analysis of the western blots was performed using ImageJ with no background subtraction. Linear fitting of data was performed in GraphPad Prism. The α-phosSer6 p53 antibody used was [Y179].

Notably, previous ELISA quantification of p53 in unstressed cells estimates the intracellular concentration of p53 to be 0.06-6 μM (monomer).[6] Following activation, p53 is estimated to increase 3-10 fold inside the cells.[7] Therefore, assuming 1×10^6 cell loading on a typical western blot, we estimate that on average about 1-15 ng of p53 could be detected from unstressed cells, and 15-150 ng of p53 could be isolated in its activated form. Therefore, the western blots and the dynamic range of detection we demonstrate, are reflective of a typical experimental set-up.

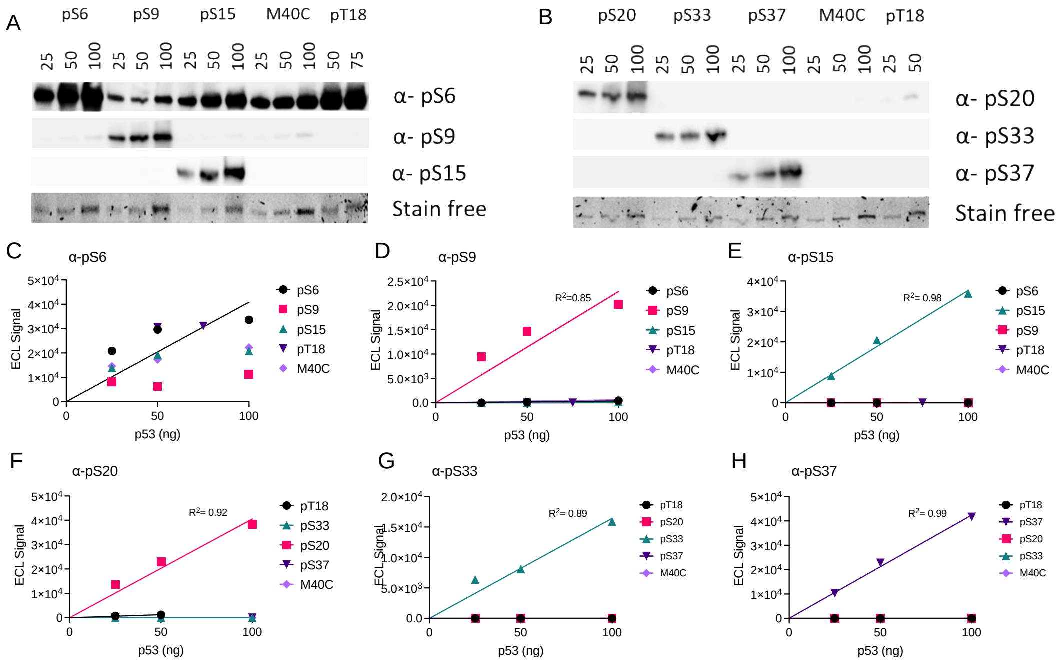

Figure S26. Quantitative western blot analysis of antibody binding to p53 phospho-protein isoforms. The antibodies used and conditions are specified in Table S2. The α-phosSer6 p53 antibody used was [Y179]. The densitometric analysis was performed in ImageJ. 'M40C' refers to recombinant p53 carrying the M40C mutation. (A) Western

Blot image titrating p53 modified at pS6, pS9 or pT18 against their respective antibodies as defined in Table S2. Antibody against pS6 modified p53 was Ab32132. (B) Western Blot image titrating p53 modified at pS20, pS33 or pS37 against their respective antibodies as defined in Table S2. (C)-(H) Linear fits of densitometric analysis vs protein loading for the range of antibodies used in the analysis.

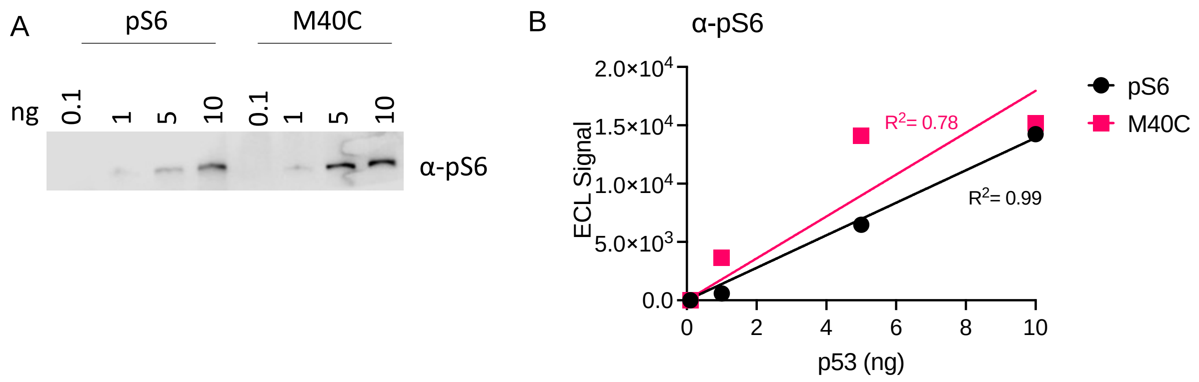

Figure S27. Evaluation of α-phosSer6 p53 antibody [Y179] specificity at lower doses of p53 confirms lack of specificity for this reagent. (A) Western blot analysis. (B) Quantification of the western blot signals from (A).

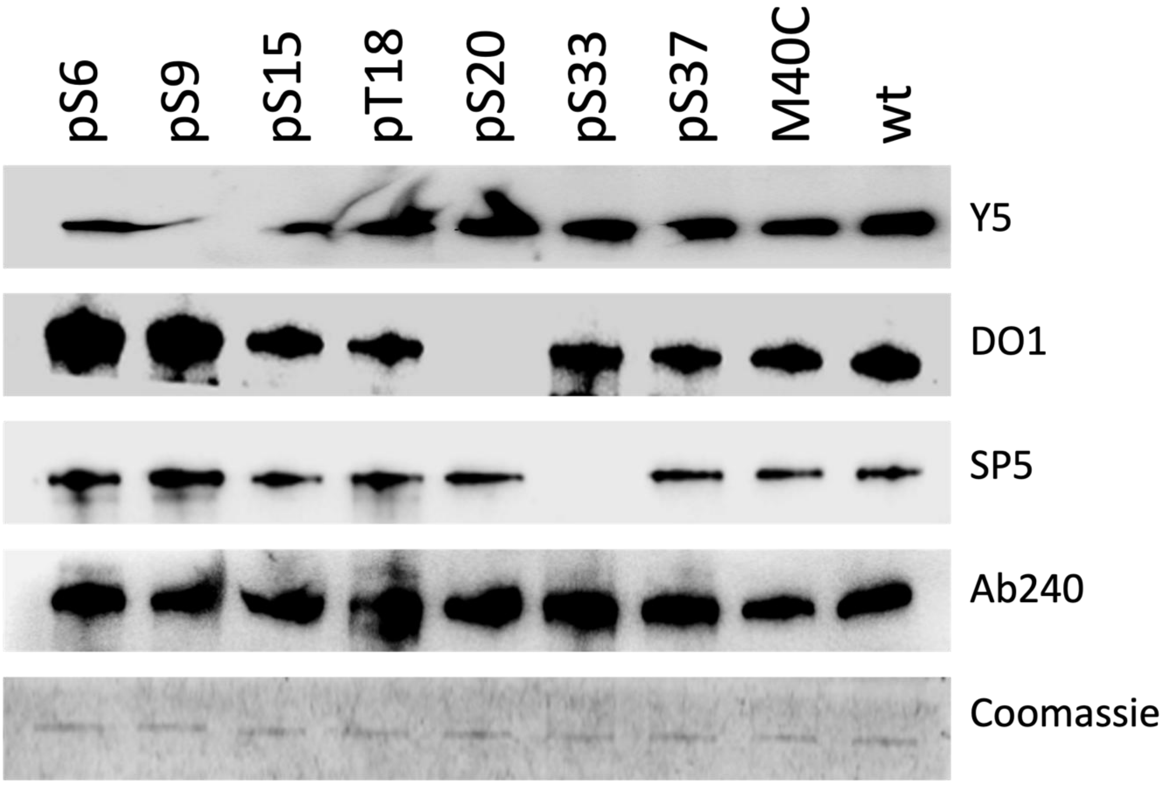

Figure S28. Western blot of 'designer' p53 library with 10 ng loading confirms specificity and biases of the α-p53 antibodies. Antibody dilutions are presented in Table S2.

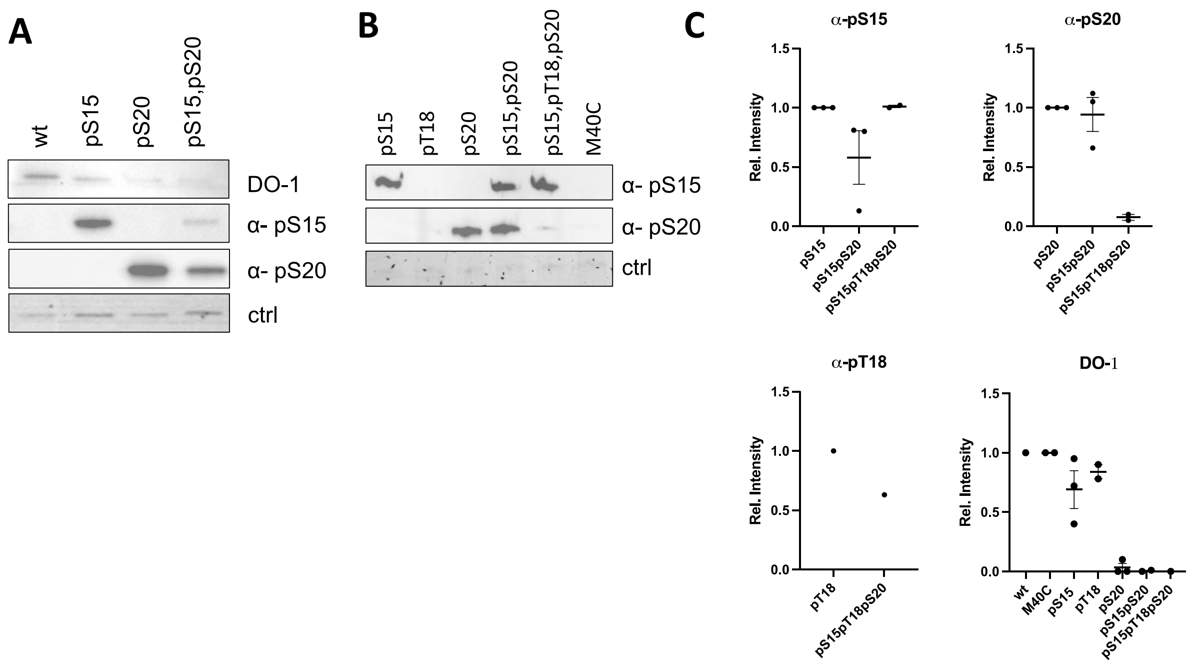

Figure S29. Epitope masking by multi-phosphorylation**.** Western blot of 'designer' p53 library with single, double and triple phosphorylation at Ser15, Thr18 and/or Ser20. (A) Analysis of epitope masking in di-phosphorylated p53 with 50 ng protein loading. (B) Analysis of epitope masking in di- and tri-phosphorylated p53 with 10 ng protein loading. (C) Densitometric analysis of Western Blot images presented in this Figure and Figure 2C. Mean and standard error of the mean shown in the Figure (n = 3 for α-pS15, α-pS20 and DO-1; n=1 for α-pT18). Relative Intensity was calculated as the ratio of band intensities of the sample compared to the signal derived from the mono-phosphorylated target variant.

Supplementary references

(1) Margiola, S.; Gerecht, K.; Müller, M. M. Semisynthetic 'Designer' P53 Sheds Light on a Phosphorylation–Acetylation Relay. *Chem. Sci.* **2021**. https://doi.org/10.1039/D1SC00396H.

(2) Kuipers, B. J. H.; Gruppen, H. Prediction of Molar Extinction Coefficients of Proteins and Peptides Using UV Absorption of the Constituent Amino Acids at 214 Nm To Enable Quantitative Reverse Phase High-Performance Liquid Chromatography−Mass Spectrometry Analysis. *J. Agric. Food Chem.* **2007**, *55* (14), 5445–5451. https://doi.org/10.1021/jf070337l.

(3) Ferrige, A. G.; Seddon, M. J.; Jarvis, S.; Skilling, J.; Aplin, R. Maximum Entropy Deconvolution in Electrospray Mass Spectrometry. *Rapid Commun. Mass Spectrom.* **1991**, *5* (8), 374–377. https://doi.org/10.1002/rcm.1290050810.

(4) Minkovich, B.; Ruderfer, I.; Kaushansky, A.; Bravo-Zhivotovskii, D.; Apeloig, Y. α-Sila-Dipeptides: Synthesis and Characterization. *Angew. Chem. Int. Ed.* **2018**, *57* (40), 13261–13265. https://doi.org/10.1002/anie.201807027.

(5) Bell, S.; Hansen, S.; Buchner, J. Refolding and Structural Characterization of the Human P53 Tumor Suppressor Protein. *Biophys. Chem.* **2002**, *96* (2), 243–257. https://doi.org/10.1016/S0301-4622(02)00011-X.

(6) Ma, L.; Wagner, J.; Rice, J. J.; Hu, W.; Levine, A. J.; Stolovitzky, G. A. A Plausible Model for the Digital Response of P53 to DNA Damage. *Proc. Natl. Acad. Sci. U. S. A.* **2005**, *102* (40), 14266. https://doi.org/10.1073/pnas.0501352102.

(7) Harris, S. L.; Levine, A. J. The P53 Pathway: Positive and Negative Feedback Loops. *Oncogene* **2005**, *24* (17), 2899–2908. https://doi.org/10.1038/sj.onc.1208615.
